# Multi-omics analysis to reveal the synergistic mechanism underlying the multiple ingredients of *Stephania tetrandra* extract on rheumatoid arthritis through the PI3K/Akt signaling pathway

**DOI:** 10.3389/fphar.2024.1447283

**Published:** 2024-08-16

**Authors:** Jinfeng Chen, An Zhang, Anzheng Nie, Xiaoxiao Zuo, Lei Zhang, Yuxue Jiao, Lulu Wang, Yang Yang, Kun Liu, Xinli Xue, Yuanyuan Zhuang, Yansha Meng, Jing-Hua Yang

**Affiliations:** ^1^ Research Center for Clinical Systems Biology, Translational Medicine Center, The First Affiliated Hospital of Zhengzhou University, Zhengzhou, Henan, China; ^2^ Institute of Infection and Immunity, Henan Academy of Innovations in Medical Science, Zhengzhou, Henan, China; ^3^ Department of Traditional Chinese Medicine, The First Affiliated Hospital of Zhengzhou University, Zhengzhou, Henan, China; ^4^ Radiotherapy Department, The First Affiliated Hospital of Zhengzhou University, Zhengzhou, Henan, China; ^5^ Department of Pharmacy, The First Affiliated Hospital of Zhengzhou University, Zhengzhou, Henan, China

**Keywords:** *Stephania tetrandra*, multi-omics joint analysis, rheumatoid arthritis, PI3K/Akt pathway, synergistic mechanism, abatacept therapy

## Abstract

**Background:** *Stephania tetrandra* has been used for treating rheumatic diseases for thousands of years in rural areas of China. Several studies have found that tetrandrine and fangchinoline can inactivate the PI3K/Akt signaling pathway by reducing the expression and phosphorylation of AKT. However, the mechanism underlying the therapeutic actions of *S. tetrandra* on RA is not well known.

**Methods:** In this study, we determined the molecular mechanism of the therapeutic effects of the multiple ingredients of *S. tetrandra* extract (STE) on collagen-induced arthritic (CIA) rats by integrating pharmacometabolomics, proteomics, and PTMomics.

**Results:** In the multi-omics joint analysis, first, the expression signatures of proteins, PTMs, metabolites, and STE ingredients were profiled in CIA rats PBMCs that underwent STE treatment. Bioinformatics analysis were subsequently probed that STE mainly regulated tryptophan metabolism, inflammatory response, and cell adhesion pathways in CIA rats. The interrelated pathways were further constructed, and the findings revealed that STE attenuated the inflammatory response and proliferation of PBMCs in CIA rats by mediating the key targets of the PI3K/Akt pathway, including Hint1, ACP1, FGR, HSP90@157W + dioxidation, and Prkca@220N + 845.4540 Da. The rheumatic functions of Hint1 and ACP1 were further confirmed by applying a transcriptomic data of RA patients who clinically received abatacept therapy. Furthermore, a cross-ome correlation analysis was performed and major *in vivo* ingredients of STE, including coclaurine-*N*-glucuronide, Me,coclaurine-*O*-glc, *N*-gluA-schefferine, corydamine, corypamine, tetrandrine, and fangchiniline, were found to act on these targerts to inactivate the PI3K/Akt pathway.

Conclusion: These results elucidated the molecular mechanism by which the ingredients of STE mediate the expression of the key targets in the PI3K/Akt pathway, leading to anti-rheumatic functions. The findings of this study provided new insights into the synergistic effect of STE against arthritis in rats.

## 1 Introduction

Rheumatoid arthritis (RA) is a chronic and systemic autoimmune inflammatory disorder affecting multiple tissues and organs, especially flexible joints. Systemic inflammation, proliferation, autoantibodies, synovial hyperplasia, and erosion of the bone and cartilage are the primary characteristics of RA. Although many patients can now attain disease remission, precision medicine for RA and the prevention and cure of RA still need to be further investigated (Di Matteo et al., 2023).

*Stephania tetrandra* radix, the roots of *S. tetrandra* S. Moore (Menispermaceae), has been used for treating rheumatic diseases for thousands of years in rural areas of China and has shown promising immunomodulatory effects in the treatment of RA (Zhang et al., 2020). The known anti-inflammatory ingredients of *S. tetrandra*, tetrandrine and fangchinoline, can also effectively prevent tumorigenesis (Xu et al., 2021; Zhang et al., 2021). Fangchinoline can inhibit cell proliferation, induce cell cycle arrest, and promote apoptosis by suppressing the phosphatidylinositol 3-kinase/protein kinase B (PI3K/Akt) and mitogen-activated protein kinase (MAPK) signaling pathways (Shao et al., 2022). A study found that tetrandrine ameliorated RA in mice by inhibiting the migration and invasion of RA fibroblast-like synoviocytes (FLS) through activating the PI3K/Akt and JNK signaling pathways (Lv et al., 2015). These studies found that tetrandrine and fangchinoline can regulate RA to some extent through the PI3K/Akt signaling pathway. However, the mechanism of action of a single ingredients cannot explain the overall mechanism underlying the therapeutic effects of *S. tetrandra* on RA, since there are various isoquinolines in *S. tetrandra* (Chen et al., 2020), which can also regulate cell proliferation and inflammation, similar to tetrandrine and fangchinoline (Li et al., 2023).

Peripheral blood represents the main medium for the transport of components of the immune system. Peripheral blood mononuclear cells (PBMCs), are the immune cells that initiate the autoimmune inflammatory process against target organs (Wang et al., 2022). Pro-inflammatory cytokines, such as interleukin (IL)-1β, IL-6, and tumor necrosis factor-α (TNF-α), can activate the PI3K/Akt signaling pathway, which can lead to the upregulation of the expression of effector proteins, such as F-actin, and then, induce a proliferative response in PBMCs (Vicente-Manzanares and Horwitz, 2011; Profumo et al., 2013). These findings suggest that the association between the inflammatory response and migration of PBMCs may be the important effectors responsible for the pathophysiological processes of RA. Considering that tetrandrine can mediate immunosuppression in human peripheral blood T cells by downregulating the IkB kinase-IkBα-NFkB signaling pathway (Ho et al., 2004), the molecular expression signatures of PBMCs altered by *S. tetrandra* can reveal the mechanism underlying the therapeutic effects of tetrandrine on RA.

**FIGURE 1:**
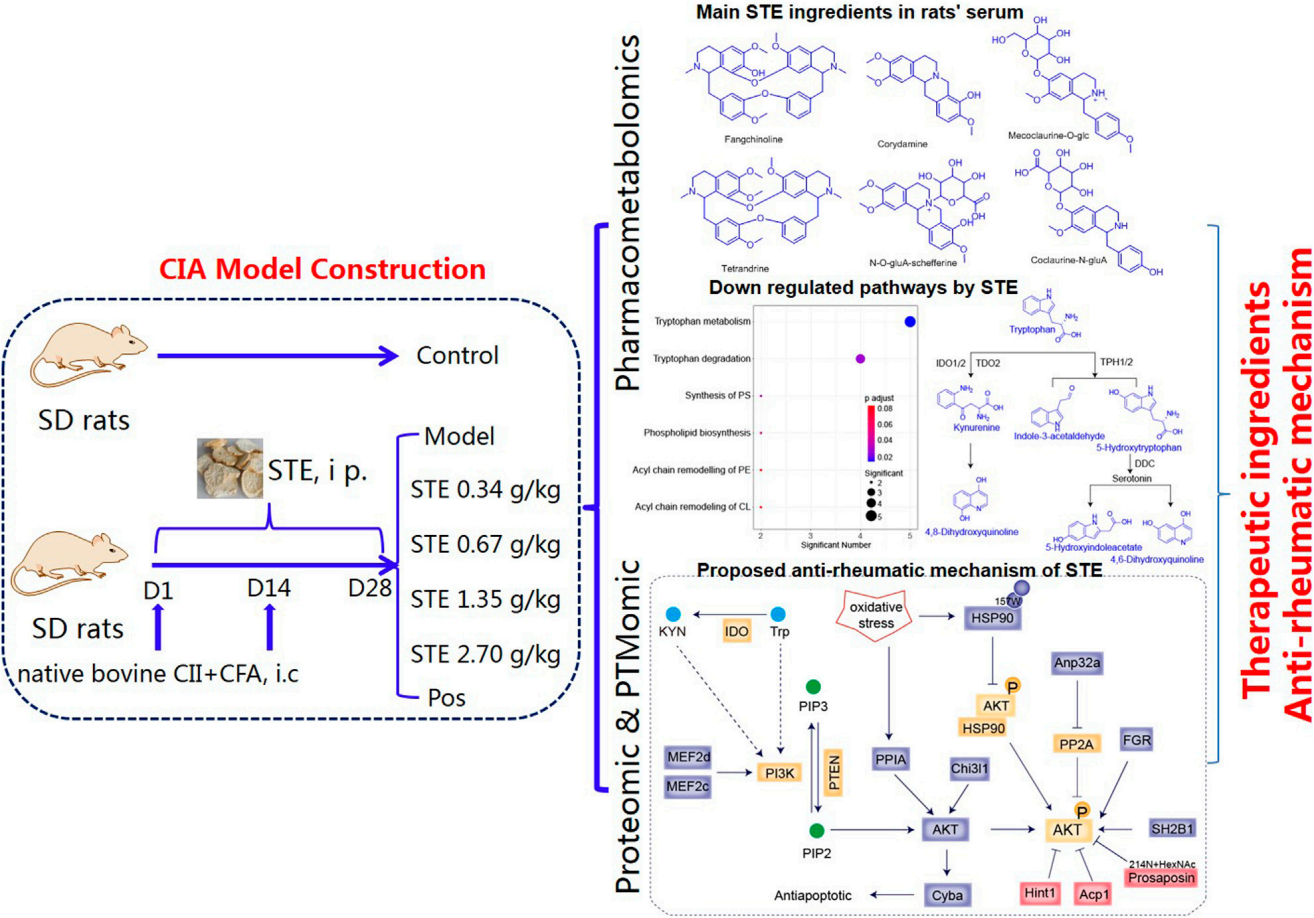
Overview of the sample cohort and experimental design to elucidate the mechanism underlying the regulatory effects of *Stephania tetrandra* extract on the pathogenesis of rheumatoid arthritis, through integration of pharmacometabolomics, proteomics, and PTMomics.

In this study, pharmacometabolomics, proteomics, and PTMomics techniques were integrated to provide a system-wide understanding of the molecular mechanisms underlying the therapeutic effects of *S. tetrandra* extract (STE) on rats with collagen-induced arthritis (CIA, Figure 1). By conducting an cross-ome correlation analysis, we aimed to gain new insights into the functional interactions or co-regulation between various active ingredients of STE and different classes of biomolecules. Using this approach, we found that interactions occurred at the pharmacometabolome, proteome, and PTM levels and established a reference framework for future research.

## 2 Materials and methods

### 2.1 Chemicals and Regents

Incomplete Freund’s adjuvant (inCFA), complete Freund’s adjuvant (CFA), and Bovine Collagen Type II (Bornstein and Traub Type II, powder) were purchased from Sigma-Aldrich (St. Louis, MO, United States). Ultra-pure water (18.2 MΩ at 25°C) was prepared in-house using a GenPure UV/UF water purification system (Thermo Fisher Scientific). LC-MS grade acetonitrile, methanol, and formic acid were purchased from Merck (Darmstadt, Germany). Rat Interleukin 6 (IL-6) ELISA Kit (Lot: Z22037901), and Rat Interleukin 1β (IL-1β) ELISA Kit (Lot: Z20037999) were purchased from Wuhan Huamei Biotechnology Co., Ltd.

Tetrandrine and fangchinoline were purchased from the Chengdu Must Bio-Tech Co., Ltd. (Chengdu, China). Corydalmine was obtained from ShanghaiyuanyeBio-TechnologyCo.,Ltd. (Shanghai, China). Their purities were determined to be greater than 98% by high performance liquid chromatography (HPLC) analyses. *S. tetrandra* extract (STE), processed from the dried root of *S. tetrandra* S. Moore (Menispermaceae), was purchased from Lanzhou wotelaisi Biotechnology Co., Ltd. (Batch Number: 20180726, Lanzhou, China, tetrandrine, 1.34%; fangchinoline, 0.73%, Supplementary Table S1). Chemical composition of STE was profiled by LC-HRMS/MS (Supplementary Figure S1), with the assistance of the home database (Chen et al., 2020) and reference standards. Diclofenac was purchased from Jilin Yizheng Pharmaceutical Group Co., Ltd. (Jilin, China).

### 2.2 Sample cohort and experimental design

All purchases and operations of experimental animals were approved by the Animal Experiment Ethics Committee of the First Affiliated Hospital of Zhengzhou University. All experiments strictly followed the Regulations on the Management of Laboratory Animals issued by the State Science and Technology Commission (the requirement for the care and use of laboratory animals; US National Research Council, 2011). All rats were maintained under specific pathogen-free (SPF) conditions. They were housed in a 12 h-light/12 h-dark environment with 30%–77% relative humidity at 67°F–76°F, and given free access to food (standard chow) and water.

Male Sprague-Dawley rats (6–8 weeks old, weighing 180–220 g) were used in this study. The CIA model was established using a previously reported method (Brand et al., 2007). Briefly, native bovine CII was dissolved in 0.01 M acetic acid at a concentration of 2 mg/mL with gentle stirring and incubated overnight at 4°C. The solution was emulsified with the same volume of CFA. The rats in the experimental group were immunized by intradermal injection at the base of the tail, back, and hind paws with 0.1 mL of collagen emulsion. The rats in the sham control group were injected with normal saline. The rats received a booster shot 14°days later by the same method with inCFA. The body weight and the edema of the left hind paws of rats were periodically measured after the second induction. These measurements were used to assess the therapeutic effect of the drug on the affected foot.

After the first injection, the CIA rats were randomly divided into seven groups (n = 8 rats per group), including CIA model controls (Mod), vehicle controls (Con), positive controls (Pos, diclofenac, 3 mg/kg), STE-treated rats (0.34 g/kg, 0.67 g/kg, 1.35 g/kg, and 2.7 g/kg). All animals received corresponding doses *via* gastric administration daily for 28 days. The rats of the Mod and Con groups received pure water instead, and the positive controls were administered 3 mg/kg diclofenac at the same time.

All rats were anesthetized by intraperitoneal injection of 10% chloral hydrate (0.3 mL/100 g). Blood samples were collected from the abdominal aorta of the anesthetized rats in clean test tubes with or without EDTA. Then, the blood samples without EDTA were immediately centrifuged at 3,000 rpm for 10 min, and the serum samples were separated and stored at −80°C until analysis. The blood samples in EDTA anticoagulant tubes were processed to isolate PBMCs, using Ficoll-Paque PLUS solution (GE Healthcare Bioscience, Marlborough, MA, United States).

### 2.3 Histological examination

To conduct histological analysis, the rats ankle joints were dissected and immediately fixed in 10% formalin for 3 days, and then, they were decalcified in 10% EDTA, followed by embedding in paraffin for 3 weeks. Hematoxylin and eosin (H&E) were used to stain the tissue sections. The synovial tissue sections were scanned using a PANNORAMIC Midi II scanner (3DHISTECH, Budapest, Hungary). The histopathological characteristics of the joints were evaluated based on the changes in inflammatory cells, synovial hyperplasia, pannus formation, and erosion of the cartilage and bone.

### 2.4 Enzyme-linked immunosorbent assay (ELISA)

Samples were prepared from the serum of the rats. The levels of IL-1β and IL-6 in serum of rats were determined by ELISA using commercial kits according to the manufacturer’s protocol.

### 2.5 Untargeted metabolomics and lipidomics analysis

#### 2.5.1 Sample preparation for metabolomics and lipidomics analysis

First, 50 µL of serum was mixed with 200 µL of a chilled chloroform:methanol (2:1) mixture, using a mixer mill at a frequency of 30 s^−1^ for 2 min. Then, the mixture was centrifuged at 4°C for 2 min at 12,000 rpm, resulting in a biphasic extraction mixture.

For metabolomic analysis, 50 µL of the top layer of the biphasic extraction was pipetted into a 1.5 mL Eppendorf tube containing 150 µL of acetonitrile to precipitate proteins. This mixture was centrifuged at 4°C for 10 min at 12,000 rpm. A fraction of the supernatant (30 µL) was injected for the LC-MS/MS analysis, using T3 and BEH columns. Whereas, another fraction of the supernatant (30 µL) was transferred to the fresh Eppendorf tubes and dried by vacuum concentration. The dried extract was reconstituted in 30 µL of mixture solvent (water with 10 mM ammonium acetate) for hydrophilic interaction liquid chromatography (HILIC)-MS/MS analysis.

For lipidomic analysis, 100 µL of the bottom layer of the biphasic extraction was pipetted into a 1.5 mL Eppendorf tube, dried with nitrogen. The samples were redissolved with 100 µL of 0.1% formic acid in ACN:H_2_O (60:40, *v*/*v*) and centrifuged at 12,000 rpm for 15 min at 4°C. The supernatant was extracted for CSH chromatography-MS/MS analysis.

#### 2.5.2 LC-MS/MS-based metabolomics and lipidomics analysis

LC-MS/MS analyses were performed using a Vanquish UHPLC system coupled with an Orbitrap Fusion Lumos Mass Spectrometer by a Vanquish Split Sampler HT autosampler, using a Vanquish Binary Pump (Thermo Fisher Scientific).

To capture serum metabolic characteristics comprehensively, four types of chromatographic columns were used for untargeted metabolomics analysis. The ACQUITY UPLC™ T3 C_18_ column (50 mm × 2.1 mm, 1.7 μm, Waters) was used in the positive mode. The column temperature was set at 40°C, the injection volume was 1 μL, and the flow rate was 0.3 mL/min. The optimal mobile phase consisted of water containing 0.01% formic acid (A) and acetonitrile containing 0.01% formic acid (B). The optimized UPLC elution conditions were as follows: 0–6 min, 2%–45%; 6–8 min, 45%–95%. The ACQUITY UPLC™ BEH C_18_ column (50 mm × 2.1 mm, 1.7 μm, Waters) was used in the negative mode. The column temperature was set at 45°C, the injection volume was 5 μL, and the flow rate was 0.3 mL/min. The optimal mobile phase consisted of water containing 0.01% formic acid (A) and acetonitrile containing 0.01% formic acid (B). The optimized UPLC elution conditions were as follows: 0–1.5 min, 5%–30% A; 1.5–5 min, 30%–65% A; 5–6 min, 65%–95% A. The Accucore HILIC column (100 mm × 2.1 mm, 2.6 μm, Thermo) was used in the negative mode. The column temperature was set at 45°C, the injection volume was 5 μL, and the flow rate was 0.3 mL/min. The optimal mobile phase was composed of water with 10 mM ammonium acetate (A) and ACN:H_2_O (90:10, *v*/*v*) with 10 mM ammonium acetate (B). The optimized UPLC elution conditions were as follows: 0–4.0 min, 99%–50% B; 4.0–6.0 min, 50%–50% B; 6.0–6.1 min, 50%–99% B; 6.1–11.0 min, 99%–99% B. The ACQUITY UPLC™ CSH C_18_ column maintained at 50°C (50 mm × 2.1 mm x 1.7 µm; Waters) was applied for analyzing lipids in the positive mode. Mobile phase A was 0.1% formic acid in ACN:H_2_O (60:40, *v*/*v*) with 10 mM ammonium formate, and mobile phase B was 0.1% formic acid in IPA:ACN (90:10, *v*/*v*) with 10 mM ammonium formate. The optimized UPLC elution conditions were as follows: 0–2.0 min, 20%–43% B; 2.1–5.0 min, 43%–45% B; 5.0–21 min, 45%–45% B; 21–23 min, 45%–99% B. All columns were re-equilibrated with the elution conditions for 5 min before the next injection.

The LC system was coupled with an Orbitrap Fusion Lumos Mass Spectrometer (Thermo Fisher Scientific) through a heated electrospray ionization (HESI II) source (Thermo Scientific). The source conditions were as follows: the HESI II and capillary temperature was 275°C, sheath gas flow rate was 30 units, aux gas flow rate was 6 units, sweep gas flow rate was 0 units, spray voltage was |4.5 kV| for both positive and negative modes, and S-lens RF was 60.0 units. The MS was operated in a polarity switching mode acquiring positive and negative full MS and MS^2^ spectra (Top2). The acquisition parameters for full MS scans in both modes were 60,000 resolution, 1 × 10^6^ automatic gain control (AGC) target, 100 ms ion accumulation time (max IT), and 100–1,500 *m*/*z* (2,000 *m*/*z* for lipids) scan range. The MS^2^ scans in both modes were performed at 30,000 resolution, 1 × 10^5^ AGC target, 50 ms max IT, 1.0 m/z isolation window, stepped normalized collision energy (NCE) at 20, 30, and 40, and a 15.0 s dynamic exclusion.

#### 2.5.3 Metabolomics and lipidomics data processing and analysis

The LC-MS data were processed using Compound Discoverer 3.1 (Thermo Scientific) and LipiDex (Hutchins, Russell, and Coon, 2018) (v. 1.1.0). All peaks within the effective retention time and MS^1^ precursor mass of 100–1,500 Da (2,000 Da for lipids) were grouped into distinct chromatographic profiles and aligned using a 5 ppm mass and 0.3 min retention time tolerance. The profiles that did not reach a minimum peak intensity of 5 × 10^5^, a maximum peak width of 0.75, a signal-to-noise (S/N) ratio of 3, and a three-fold intensity increase over blanks were not processed further. The MS/MS spectra were searched against an *in silico* generated lipid spectral library containing 35,000 unique molecular compositions representing 48 distinct lipid classes (LipiDex library “LipiDex_HCD_Formic”, including a full range of acyl chains). The spectral matches with a dot product score greater than 500 and a reverse dot product score greater than 700 were retained for further analysis, with a minimum spectral purity of 75% for designating fatty acid composition. From the dataset, we removed adducts, class IDs greater than 3.5 median absolute retention time deviation (M.A.D. RT) of each other, and features found in less than three files. The data were also searched with Compound Discoverer 3.1 to identify metabolomics nodes for additional spectral matching with the mzCloud and mzVault libraries. The potential metabolites were identified according to the accurate m/z, retention time, and typical MS/MS fragment and pattern of the potential biomarkers by searching the HMDB (http://www.hmdb.ca/) database.

Pathway analysis was performed based on the KEGG database (http://www.genome.jp/kegg/) and the “Wu Kong” platform (https://www.omicsolution.com/wkomics/main/). The Wu Kong platform is a web-based tool used for visualizing metabolomics based on database sources, including the KEGG and the HMDB databases.

### 2.6 Proteomics and PTMomics analysis

#### 2.6.1 Sample preparation for proteomics and PTMomics analysis

First, PBMCs were homogenized with Dounce in ice-cold RIPA buffer (Millipore 20-188, United States) supplemented with 10 µL of 500 mM dithiothreitol (DTT) and 10 µL of protease inhibitor cocktail (Roche, Switzerland) to protect proteins against oxidation and degradation. After the cells were sonicated three times for 5 s on ice, the lysates were centrifuged at 12,000 rpm for 10 min at 4°C. Protein concentrations were determined using the Bicinchoninic Acid (BCA) assay (Beyond, China). Approximately 200 μg of soluble proteins were precipitated with a five-fold volume of chilled acetone. Then, the samples were incubated for at least 1 h or overnight at −80°C. After incubation, the samples were centrifuged at 4°C and 12,000 rpm for 15 min, and the supernatant was discarded. The precipitate was re-suspended in 5 mM DTT and alkylated with 15 mM iodoacetamide. The proteins were digested with sequencing grade trypsin at an enzyme-to-substrate ratio of 1:100 overnight at 37°C. The peptides were desalted with the C_18_ column (Waters) and fractionated using high-pH reversed-phase chromatography. Briefly, approximately 200 µg of the desalted peptides were re-suspended in 100 µL of ammonia water (pH 10.0) and loaded on the column filled with Xbridge Peptide BEH C_18_ (4.6 mm × 100 mm, 3.5 μm; Waters). The column was eluted with increasing concentrations of acetonitrile (6–50%) in ammonia water. Six fractions were collected, lyophilized, and stored at −80°C for further use.

#### 2.6.2 LC-MS/MS-based analysis of peptides

Peptide samples were analyzed using an EASY-nLC 1200 LC System (Thermo Fisher Scientific, Waltham, MA) coupled with a Q Exactive HF-X MS System (Thermo Fisher Scientific, Waltham, MA). The peptides were re-dissolved in mobile phase A (2% ACN and 0.1% formic acid) and directly loaded onto a reversed-phase C_18_ column (Reprosil 1.9 µm, 250xφ0.075 mm). Then, the samples were eluted with a 120-min linear gradient of 5%–35% solvent B at a flow rate of 600 nL/min (solvent A: 0.1% formic acid in water; solvent B: 0.1% formic acid in acetonitrile). The flex nanospray was used in the positive mode, and the spray voltage was set to 2.10 kV using stainless steel emitters. The transfer capillary was maintained at 300°C. Mass spectrometry was performed using a data-dependent acquisition mode. For the MS^1^ full scan, ions with *m*/*z* of 350–1,800 were acquired using an Orbitrap mass analyzer at a high resolution of 60,000. The automatic AGC was set as 3 × 10^6^. The maximal ion injection time was 50 ms. Twenty MS^2^ scans were collected after each full scan. Higher-energy collisional dissociation (HCD) was used as the MS^2^ activation type, and the resolution spectrum for HCD was set to 15,000 at 200 *m*/*z*. The normalized collision energy (NCE) for HCD was 28. Fragment ions were analyzed using an ion trap mass analyzer with AGC set as 5 × 10^4^. The maximal ion injection time of MS^2^ was 45 ms and the dynamic exclusion was 50 s.

#### 2.6.3 Proteomics data processing and analysis

The mass spectrometric data obtained were analyzed using Proteome Discoverer (Version 2.4.1.15, Thermo Fisher Scientific). The *Rattus norvegicus* fasta dataset was downloaded from UniProtKB in April 2020, which contained 8,177 reviewed protein sequences. The enzyme was set to trypsin with two missed cleavage tolerance. Static modifications were set to carbamidomethylation (+57.0215 Da) of cysteine, and variable modifications were set to oxidation (+15.9949 Da) of methionine and acetylation (+42.0106 Da) of the N-termini of peptides. The precursor ion mass tolerance was set to 10 ppm, and the product ion mass tolerance was set to 20 ppm. The peptide-spectrum match allowed 1% target false discovery rate (FDR) (strict) and 5% target FDR (relaxed). Normalization was performed against the quantity of total peptides. For the other parameters, the default setup was used. Other post-translational modifications were analyzed following a specific procedure.

#### 2.6.4 PTMomics data processing and analysis

All raw files of shotgun proteomics were compiled to analyze against the UNIPROT database (8177 entries, *R. norvegicus* proteome, April 2020) by a wildcard open search using the Byonic software (v3.8) (Bern et al., 2012). The tolerance was set to 5 ppm for precursor ions, and 20 ppm for the fragment ions. Two missed cleavages were allowed for trypsin digestions. The delta masses between coding and the observed amino acids were set from −500 to 1,000 Da, filtered with Score ≥300, Delta Mod Score ≥10, FDR 2D ≤ 0.01. The nonzero mass shifts-containing protein sites were subsequently analyzed by the Bayesian Information Criterion (BIC) and followed Gaussian mixture model with 1 Da intervals from −500 to 1,000 Da (Chen et al., 2022). The clustered peaks with the expected delta masses and sufficient values of the goodness-of-fit (R^2^) were identified as the qualified mass differences between the coding and the observed amino acids, which were considered different modifications or amino acid variations.

The latest GO database (https://www.ebi.ac.uk/QuickGO/) was used to perform the gene ontology enrichment analysis. For functional enrichment analysis, the lists of interesting proteins were submitted for enrichment analysis using all the proteins detected in the experiment as a background dataset. All terms displayed had a *p*-value below 0.05.

### 2.7 Multivariate statistical analysis

Principal component analysis (PCA) was performed using log2-transformed abundance measurements from samples containing lipids and small-molecule measurements. The R statistical and graphing environment (R version 3.6.1) and the function prcomp were used for the analysis. Using this method, the log2-transformed data were scaled to a mean equal to zero before performing PCA.

The differences between groups were determined by two-sided Student’s t-tests or Mann-Whitney U tests, whichever was suitable. The differences among the four groups (1.35 g/kg STE, Con, Mod, and Pos) were determined by conducting a one-way analysis of variance (one-way ANOVA), using the R statistical and graphing environment (R version 3.6.1). The correlation matrices were determined by conducting Spearman’s correlation coefficient, and the results were considered to be statistically significant at *p* < 0.05. The correlation networks were visualized and analyzed using the Cytoscape software (Shannon et al., 2003).

## 3 Results

### 3.1 Effect of STE on the histopathological changes in the ankle joint

**FIGURE 2:**
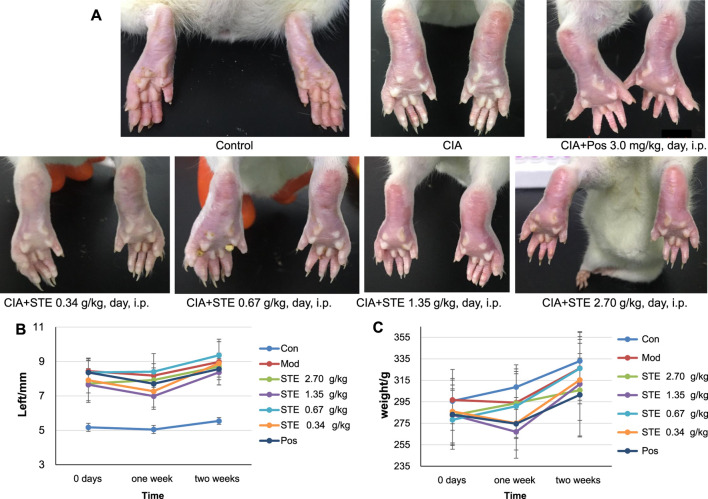
Collagen treatment induced rheumatoid arthritis (CIA) in rats, and *Stephania tetrandra* extract (STE) treatment alleviated the symptoms related to CIA. **(A)** Representative photographs of the hind paws of rats at day 28 after treatment with 1) Control (Con), 2) CIA rats, 3) positive control (Pos, diclofenac, 3 mg/kg/day i p.), 4) STE (0.34 g/kg/day i p.), 5) STE (0.67 g/kg/day i p.), 6) STE (1.35 g/kg/day i p.), and 7) STE (2.70 g/kg/day i p.). **(B)** The thickness of left hind paw in the Con, CIA, STE, and Pos rats. **(C)** Weights of Con, CIA, STE, and Pos rats. Data are presented as means ± SD (n = 7–8 per group).

Unlike the rats in the control group, the CIA rats showed dull hair, sluggish movement, and a lack of motivation. The anterior foot joints of CIA rats were red and swollen (Figure 2A). The degree of paw swelling of the CIA rats was considerably greater than that recorded in the control rats (Figure 2B), along with an abnormal increase in weight with time (Figure 2C). The degree of paw swelling was lower after treatment with STE and diclofenac, compared to that recorded in the model group. From day 14, the effect of STE (1.35 g/kg) appeared to be more significant than the effect of diclofenac on the degree of paw swelling.

**FIGURE 3:**
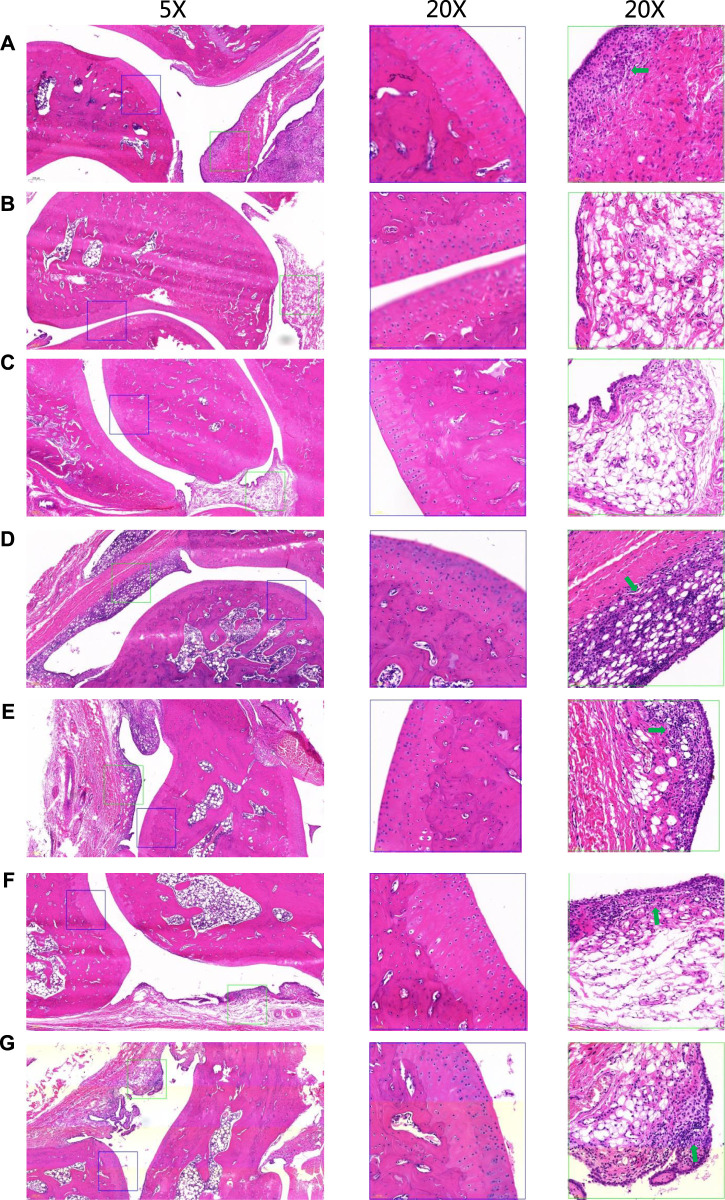
Representative hematoxylin and eosin-stained sections of the ankle joints of rats from different groups. Low-magnification images (×5) are shown on the left, and higher-magnification images (20x) of cartilage and synovial regions enclosed in blue and green boxes are separately shown in the middle and on the right. There is severe inflammatory infiltrations (green arrows). **(A)** CIA model. **(B)** Control. **(C)** Positive control (diclofenac, 3 mg/kg, day i p.). **(D)** *Stephania tetrandra* extract (STE, 0.34 g/kg, day p.). **(E)** STE (0.67 g/kg, day i p.), **(F)** STE (1.35 g/kg, day i p.), and **(G)** STE (2.70 g/kg, day i p.).

To evaluate the anti-inflammatory effect of STE, the ELISA and immunohistochemistry analysis were applied. The ELISA results indicated that the levels of IL-1β, and IL-6 in serum (Supplementary Figure S2) of CIA rats were significantly increased in model group compared with the normal rats, and decreased in groups treated with STE and diclofenac compared with the model group. The effect of STE (1.35 g/kg) appeared to be more significant than other dosages on the levels of IL-1β and IL-6. The overall pathological changes in the ankle joint of CIA rats (Figure 3A) exhibited cartilage erosion, synovial lining layer hyperplasia, disorganized arrangement of synovial cell layers, irregular arrangement of cells, and infiltration of a large number of mononuclear cells in the surrounding tissue, compared to the ankle joints of the Con rats (Figure 3B). Histopathological lesions improved in the STE-treated groups (Figures 3C–G) to different degrees, demonstrating the immunosuppressive effect of STE on CIA rats. The above results indicated that the rats in the group administered STE at the dosage of 1.35 g/kg exhibited a remarkable reduction in all pathological damages compared to the CIA rats.

### 3.2 Pharmacometabolomics analysis

**FIGURE 4:**
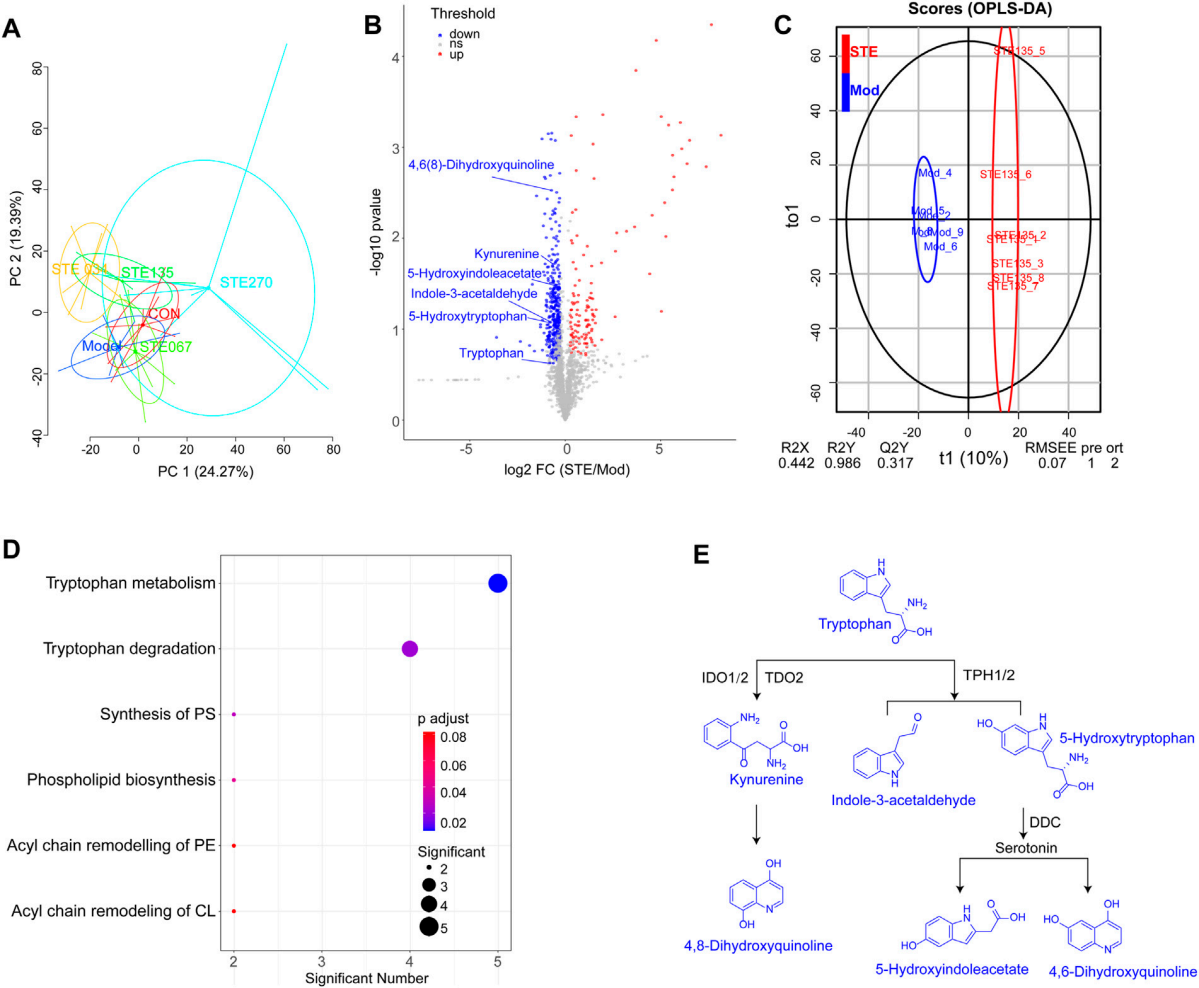
KEGG dysregulated in metabolomic by *Stephania tetrandra* extract (STE, 1.35 g/kg, day (i) p.). **(A)** PCA score plot (PC1 versus PC2) of test samples shows principal components 1 and 2 capture 24.27% and 19.39% of the variance between samples. **(B)** OPLS-DA models presented excellent classification and prediction ability of STE and CIA rats. **(C)** Rheumatoid arthritis-associated metabolites in serum were identified by conducting a volcano plot analysis. Blue and red dots represent downregulated and upregulated metabolites (STE vs. Mod). **(D)** KEGG enrichment of the downregulated metabolites in rats’ serum. **(E)** Metabolic pathways of tryptophan metabolism.

The basic peak chromatograms (BPCs) of serum metabolites are shown in Supplementary Figure S3. The results of PCA (Supplementary Figure S4A) showed that the QC samples were tightly clustered, which indicated that the results had negligible differences and the instrument was stable. The PCA results showed that after 28 days of treatment with 1.35 g/kg STE, the metabolomic profiles of rats serum shifted toward that of the control group (Figure 4A). These results also indicated that the symptoms of arthritis decreased considerably after administering 1.35 g/kg STE compared to the symptoms recorded in the CIA rats.

By comparing the metabolomic profiles of rats serum in the STE (1.35 g/kg) group with CIA rats, potential metabolites involved in the RA pathogenesis were screened based on predetermined rules (FC ≥ 1.5, *p* < 0.05, VIP ≥ 1.5; Figures 4B, C). The potential pathways affected by STE are shown in Figure 4D; Supplementary Figure S4B (Supplementary Table S2). Tryptophan metabolism was a significantly dysregulated pathway in which the levels of six metabolites decreased, including tryptophan, 5-hydroxytryptophan, kynurenine, 5-hydroxyindoleacetate, 4,6(8)-dihydroxyquinoline, and indole-3-acetaldehyde (Supplementary Figure S5). The metabolomic results were consistent with that tryptophan metabolism was reduced in the sera of RA patients (Kang et al., 2015).

The majority of tryptophan (∼99%) is metabolized along the kynurenine pathway (Figure 4E). Tryptophan, the rarest essential amino acid, may be associated with the development of arthritis through its effects on the activation of CD4^+^ T cells and the production of autoantibodies (Shime et al., 2008). Its metabolite, kynurenine has an immunomodulatory function and exerts immunosuppressive effects (Panfili et al., 2020). Some studies found that 5-hydroxytryptophan, an intermediate in the serotonin synthesis process, could not only suppress the activation of p38 and nuclear factor-κB (NF-κB) in fibroblasts (Bae et al., 2010) but also inhibit the production of pro-inflammatory cytokines (Yang et al., 2015). Thus, the downregulation of tryptophan metabolism may prevent inappropriate immune responses from being elicited in arthritic joints. These findings indicated that STE may perform its anti-inflammatory activity by regulating the metabolic levels of tryptophan, kynurenine, and 5-hydroxytryptophan.

### 3.3 Determining the potential anti-rheumatic mechanism of STE

**FIGURE 5:**
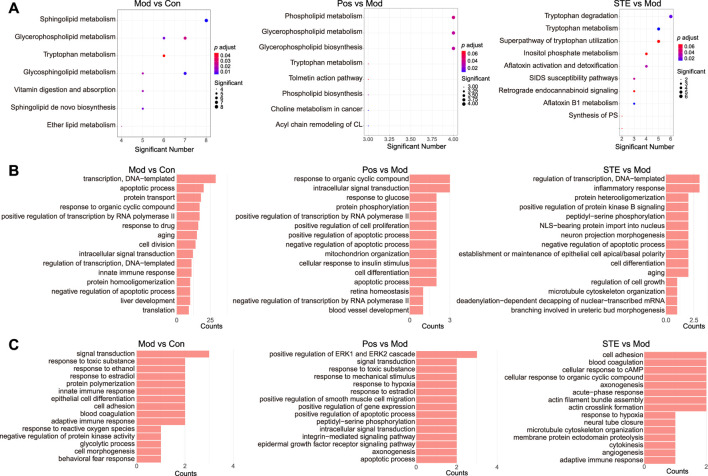
The results of the multi-omics analysis showed that strong molecular signatures were associated with CIA and *Stephania tetrandra* extract. **(A)** KEGG enrichment of the dis-regulated metabolites in rats’ serum. **(B)** Gene ontology (GO) enrichment of the dis-regulated proteins in rats’ PBMC by proteomic analysis. **(C)** GO enrichment of the dis-modified proteins in rats’ PBMC by PTMomic analysis.

The metabolic differences between STE and diclofenac (Supplementary Figure S4A) in the serum metabolome revealed the differences in the mechanisms underlying their anti-rheumatic effects. ANOVA of the multiple omics were performed to identify the potential anti-rheumatic mechanism of STE. The major disturbed metabolic pathways enriched in the serum of CIA rats compared to those in the control rats included sphingolipid metabolism, glycerophospholipid metabolism, and tryptophan metabolism (Figure 5A). The intervention of STE and diclofenac reversed these changes in different ways. Diclofenac mainly regulated the pathways related to phospholipid metabolism and glycerophospholipid metabolism, whereas STE mainly regulated tryptophan metabolism. The disturbances in proteins and PTMs by STE and diclofenac were attributed to their effects on different biological processes. In diclofenac-treated rats, the differentially expressed proteins were associated with the biological processes of response to organic cyclic compounds, intracellular signal transduction, and response to glucose (Figure 5B; Supplementary Table S3). In contrast, the proteins regulated by STE were predominantly involved in the regulation of the biological processes of transcription, inflammatory response, and protein heterodimerization. In diclofenac-treated rats, the regulated PTMs were attributed to the positive regulation of the biological processes of the extracellular signal-regulated kinase (ERK)1 and ERK2 cascades, signal transduction, and response to toxic substance (Figure 5C). The PTMs regulated by STE mainly participated in the biological processes of cell adhesion, blood coagulation, and cellular response to cAMP. To summarize, STE may exert its anti-rheumatic effects by influencing the pathways of tryptophan metabolism, inflammatory response, and cell adhesion.

### 3.4 The mechanism underlying the anti-rheumatic effects of STE

**FIGURE 6:**
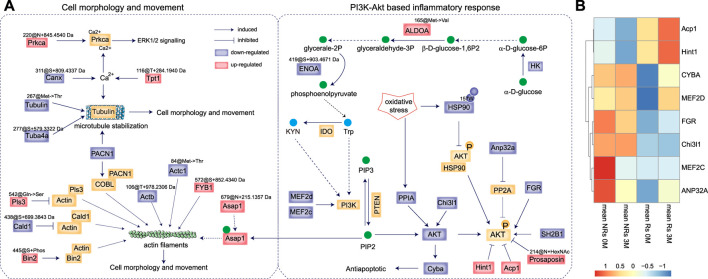
The anti-rheumatic targets of *Stephania tetrandra* extract (STE). **(A)** The proposed mechanism of action by which STE inhibited inflammatory and proliferative response in PBMCs and reduced the pathogenesis of rheumatoid arthritis. **(B)** The unsupervised clustering analysis of RNA expression of the target proteins in the PBMCs of rheumatoid arthritis patients before and after accepted abatacept therapy. “Rs” indicates responders, “NRs” indicates non-responders; “0 M” indicates pre-treatment, and “3 M” indicates 3 months post-treatment.

To elucidate the mechanism underlying the anti-rheumatic effects of STE, the regulated proteins, metabolites, and PTMs in the tryptophan metabolism, inflammatory response, and cell adhesion pathways were further analyzed to construct a network (Figure 6A). The results revealed that most molecules were associated with the regulation of the PI3K/Akt-based inflammatory and proliferative responses of PBMCs.

The decrease in the expression of tryptophan and kynurenine by STE rapidly inactivated PI3K/Akt signaling (Bishnupuri et al., 2019), which occurred probably due to the decreased expressions of HK, ENOA@419S + 903.4671 Da, and the highly expressed of ALDOA@165Met−> Val substitution, considering that phosphoenolpyruvate can be converted into tryptophan. The proteomic results indicated that STE may decrease the level of expressions of the PI3K and AKT proteins by downregulating Myocyte-specific enhancer factor 2 (MEF2d & MEF2c) (Liu et al., 2019), Chitinase-3 like-protein-1 (Chi3l1) (Zhao et al., 2022), and peptidyl-prolyl cis-trans isomerase A (PPIA). The low expression of AKT could downregulate the Cytochrome β-245 light chain (Cyba) and then activate the anti-apoptotic pathway (Chang et al., 2009). Additionally, SH2B adapter protein 1 (SH2B1) (Wang et al., 2004) and tyrosine-protein kinase (FGR) (Lee et al., 2011) were downregulated by STE, which led to the inactivation of the PI3K/Akt pathway through the dephosphorylation of AKT. Also, the decreased expression of acidic leucine-rich nuclear phosphoprotein 32 family member A (Anp32a) acted as a negative regulator of AKT phosphorylation by counteracting the effect of PP2A in cells (Tsujio et al., 2005). We found that STE further enhanced the dephosphorylation of AKT by upregulating the protein expression of Hint1 (Wu et al., 2016) and low-molecular-weight phosphotyrosine protein phosphatase (Acp1) (Teruel et al., 2011). The decrease in the dioxidation of HSP90 at 157Trp may function as a negative regulator of AKT signaling by enhancing the binding to AKT, which can lead to the dephosphorylation and inactivation of AKT (Sato et al., 2000). As Prosaposin ablation inactivates the AKT signaling pathway (Morales and Badran, 2003), increasing the level of acetyl hexosaminated 214Asn of Prosaposin may inactivate the AKT signaling pathway.

The above results indicated STE exerted synergistic effects on RA by increasing the protein levels of Hint1 and Acp1, and decreasing the expressions of FGR, Chi3l1, and CYBA. To examine the anti-rheumatic functions of these targerts, a transcriptomic data of PBMC samples from RA patients who clinically received abatacept therapy (Iwasaki et al., 2024) were applied. The transcriptomic data showed that patients with high levels of Hint1 and Acp1, low levels of Chi3l1 and FGR will benefit from the abatacept therapy (Figure 6B). And the abatacept therapy after 3 months increased the transcriptomic levels of Hint1 and Acp1, which were consistent with their protein expressions in the PBMCs of CIA rats treated by STE, further indicating the beneficial effects of STE on RA.

The cytoskeleton is composed of actin filaments, microtubules, and intermediate filaments in the cytoplasm. It supports and maintains cell morphology and movement and acts as the key mediator for the migration and invasion of PBMCs (Profumo et al., 2013). The lower levels of Actb@106T + 978.2306 Da and Actc1@84Met- > Thr substitution may be associated with actin filaments. Alterations in actin-binding proteins, including the upregulation of the phosphorylation of bridging integrator-2 (Bin2) at 445Ser, the Q542S mutation of Plastin-3 (Pls3) (Le Clainche et al., 2010), and a decrease in the expression of +699.3843 Da at the 438 Ser of caldesmon (Cald1) (Yamashiro et al., 1995), may stabilize the structure of actin filaments (Le Clainche et al., 2010). As Asap1 can coordinate the remodeling of the actin cytoskeleton by binding to PIP2 (Muller et al., 2010), the overexpression of Asap1@679N + 215.1357 Da may also involve in regulating the actin filaments (Schwintzer et al., 2011). The upregulation of FYN-binding protein 1 (FYB1)@572S + 852.4340 Da may also function in the remodeling of actin filaments (Krause et al., 2000). A decrease in the expression of PACN1 can adversely affect the reorganization of the actin filaments *via* its interaction with COBL (Schwintzer et al., 2011), which also negatively regulates the stabilization of Tubulin, the major components of microtubules. The decreased levels of Tubulin@267Met- > Thr substitution and Tuba4a@277S + 579.3322 Da may lead to microtubule destabilization. Calcium ions (Ca^2+^) also regulate the stabilization of Tubulins, where the down-modified proteins Canx@311S + 809.4337 Da and the increased Tpt1@116T + 284.1940 Da may play roles in the stabilization of Tubulins through their effects on the binding of proteins with Ca^2+^. On the other hand, Ca^2+^ ions can activate PKC alpha (Prkca) in the signaling cascades of MAPK1/3 to mediate cell proliferation and inflammation (Braz et al., 2002). The higher production of Prkca@220N + 845.4540 Da may also be involved in the proliferation and inflammatory response of PBMCs.

To summarize, STE downregulated the protein level of AKT through decreasing the expressions of Cyba, Chi3l1 and PPIA, concurrently, stimulated the dephosphorylation of AKT by increasing the protein expressions of Hint1 and Acp1 and decreasing the protein expressions of SH2B1, FGR and Anp32a. Along with the inactive PI3K/Akt signaling pathway, several PTMs, including the increasing modifications of FYB1@572S + 852.4340 Da, Bin2@445Ser + phosphorylation, and the down-regulations of Tubulin@267Met- > Thr substitution, Actc1@84@Met- > Thr substitution were involved in the proliferation of PBMCs. Thus, STE mediated the proliferation and inflammatory response of PBMCs by inactivating the PI3K/Akt signaling pathway.

### 3.5 Synergistic mechanism of action of multiple ingredients of STE

**FIGURE 7:**
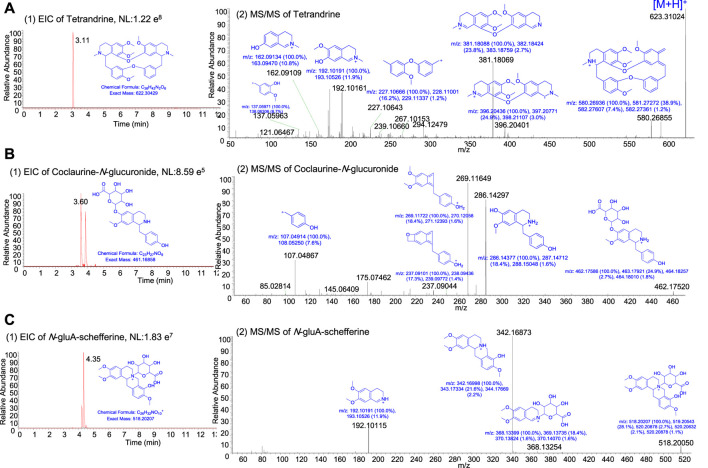
The representative extracted ion current chromatogram and MS/MS spectra of the ingredients in rats after the oral administration of *Stephania tetrandra* extract. **(A)** Tetrandrine. **(B)** Coclaurine-*N*-glucuronide. **(C)** *N*-gluA-schefferine.

According to the chemical copposition of *S. tetrandra* (Chen et al., 2020) and reference ingredients, tetrandrine, fangchinoline, corypamine, coclaurine-*N*-glucuronide, Me,coclaurine-*O*-glc, *N*-gluA-schefferine, and corydamine were identified as the major *in vivo* ingredients of STE (1.35 g/kg) after it was administered for 4 weeks (Figure 7; Supplementary Figure S6). The interrelationship pathway (Figure 6A) displayed the key molecules with strong biological associations across the PI3K/Akt pathway, regulating the inflammatory response and proliferation of PBMCs. To determine the functional connections or co-regulation between STE ingredients and the key molecules across the PI3K/Akt pathway, a pairwise cross-ome correlation analysis was conducted.

**FIGURE 8:**
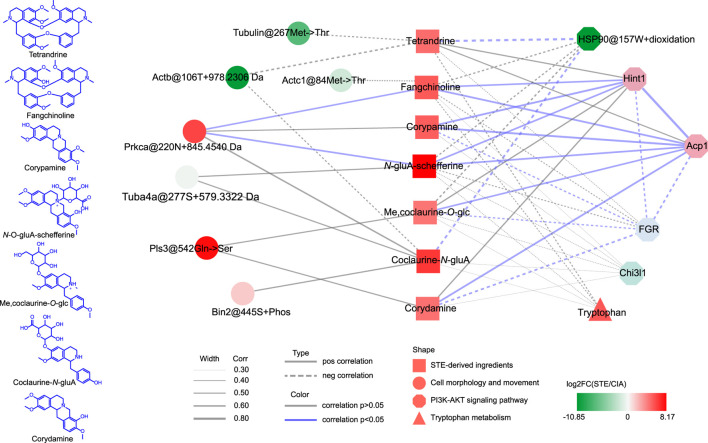
Synergistic mechanism of action of multiple ingredients of *Stephania tetrandra* extract. The statistical methods used are explained in the Materials and Methods section.

The results of the correlation analysis (Figure 8) revealed that the *in vivo* STE ingredients were significantly correlated with the key molecules in the PI3K/Akt pathway, except the serum tryptophan metabolites. To elaborate, fangchinoline, corypamine, *N*-gluA-scheffereine, Me,coclaurine-*O*-glc, and corydamine were strongly positively correlated with the protein expressions of Hint1 and Acp1. Additionally, the protein levels of Hint1 and Acp1 were significantly negatively correlated with that of FGR, which was inversely regulated by corypamine, Me,coclaurine-*O*-glc, and corydamine. Moreover, tetrandrine and coclaurine-*N*-glucuronide were strongly negatively correlated with the dioxidation level of HSP90 at 157W. The results indicated that these ingredients acted cooperatively to improve rheumatic inflammation and proliferation by inhibiting the phosphorylation of AKT and restoring the abnormal signaling of the PI3K/Akt pathway. Only Prkca@220N + 845.4540 Da was significantly positively correlated with fangchinoline and *N*-gluA-schefferine, which may participate in the regulation of cell proliferation and inflammation by activating signaling cascades involving MAPK1/3 (Braz et al., 2002).

Based on these results, the synergistic mechanism of action of multiple ingredients of STE on the key molecules of the PI3K/Akt signaling pathway may be as follows. After administering STE, tetrandrine may stimulate AKT *via* PP2A-mediated dephosphorylation by attenuating the oxidation-induced HSP90@157W + oxidation, which may inhibit the binding of HSP90 to AKT (Sato et al., 2000). Corydamine probably played a similar role, considering that it was also negatively correlated with HSP90@157W + oxidation. Fangchinoline, corypamine, and *N*-gluA-schefferine can not only stimulate the production of Hint1 but also act synergistically with diMe,coclaurine-*O*-glc and coclaurine-*N*-gluA to increase the protein level of Acp1, which can cooperatively modulate the PI3K/Akt pathway by decreasing the phosphorylation of AKT (Lee et al., 2011; Teruel et al., 2011; Wu et al., 2016). Moreover, corypamine and corydamine can further inactivate AKT by significantly decreasing the production of FGR. Furthermore, fangchinoline and *N*-gluA-schefferine can prevent the abnormal proliferation of PBMCs, caused by RA, by strongly promoting the modification of a potential new PTM with +845.4540 Da at 220N of Prkca, which may stimulate the ERK1/2 pathway.

The results of the correlation analysis complemented the interaction pathway presented in Figure 6A and indicated the synergistic therapeutic effects of the ingredients of STE acting on the key molecules of the PI3K/Akt signaling pathway.

## 4 Discussion

RA is a chronic and systemic autoimmune inflammatory disorder that affects the physical and mental health of patients. Although many patients can now achieve disease remission due to the development of novel therapeutic approaches, more information on RA pathogenesis, precision medicine approach, prevention, and cure of RA is still needed (Di Matteo et al., 2023). *Stephania tetrandra* can substantially decrease the severity of RA by controlling systemic symptoms and has been extensively used in clinical practice for several years. The PI3K/Akt signaling pathway constitutes the key mediators of RA inflammation, migration, and invasion (Vicente-Manzanares and Horwitz, 2011). Tetrandrine and fangchinoline have immunosuppressive functions and can prevent tumorigenesis by suppressing the PI3K/Akt signaling pathway (Lv et al., 2015; Yang et al., 2020; Shao et al., 2022). By using a stepwise DFI and NL-dependent structure annotation algorithm-based UHPLC-Q-TOF-MS, we identified other potential ingredients of *S. tetrandra* that can suppress key processes related to RA (Chen et al., 2020). However, the mechanism underlying the therapeutic effects of *S. tetrandra* on RA remains undetermined, as the mechanism of action of a single ingredient cannot adequately explain the overall mechanism of action of *S. tetrandra* on RA.

The association between the inflammatory response and migration of PBMCs acts as an important effector for the pathophysiological process of RA (Ho et al., 2004; Vicente-Manzanares and Horwitz, 2011; Profumo et al., 2013; Wang et al., 2022). Thus, pharmacometabolomics, proteomics, and PTMomics were integrated in this study to map the molecular expression signatures of PBMCs to provide a system-wide understanding of the molecular mechanisms underlying the therapeutic effects of *S. tetrandra* on rats with CIA. The integrated multi-omics approach not only confirmed the anti-rheumatic potential of *S. tetrandra* but also provided a better understanding of the underlying mechanisms from a global perspective. The results showed that *S. tetrandra* can improve the health of the joints of the model rats with CIA by reducing the inflammatory response and proliferation of PBMCs through regulating the PI3K/Akt pathway. The findings strongly indicated that the inactivation of the PI3K/Akt pathway is the primary mechanism by which *S. tetrandra* prevents RA-induced inflammation, migration, and invasion (Vicente-Manzanares and Horwitz, 2011; Ma et al., 2024).

Abatacept was clinically applied in treating RA under the mechanism of action involves binding to CD80 and CD86 on antigen-presenting cells, thereby inhibiting T-cell activation through blocking the costimulatory interaction with CD28 on T cells. Given that the protein expressions of Hint1 and Acp1 in the PBMCs of CIA rats treated by STE, were consistent with their transcriptomic levels in the PBMCs of RA patients after the abatacept therapy. *S. tetrandra* may play anti-rheumatic functions through inhibiting T-cell activation as abatacept did.

Several studies have shown that tetrandrine and fangchinoline can regulate RA to some extent through their effects on the PI3K/Akt signaling pathway (Yang et al., 2020; Xu et al., 2021; Zhang et al., 2021; Shao et al., 2022). Some researchers identified AKT as the target of tetrandrine and fangchinoline through molecular docking analysis (Shao et al., 2022; Ma et al., 2024). The results of Western blotting assays showed that fangchinoline and tetrandrine can suppress the expression and phosphorylation of AKT in cells (Jiang et al., 2021; Ma et al., 2024; Shao et al., 2024). However, the findings of this study suggested that AKT phosphorylation was the major molecular target of *S. tetrandra*. Additionally, fangchinoline inhibited the phosphorylation of AKT in the PBMCs of CIA rats by increasing protein expression of Hint1 and Acp1 and decreasing the expression level of FGR. Corypamine, *N*-gluA-schefferine, Me,coclaurine-O-glc, and corydamine accompanied fangchinoline in the phosphorylation of AKT; thus, they participated in the synergistic therapeutic intervention of RA. Fangchinoline also influenced ERK1/2 signaling by increasing Prkca@220N + 845.4540 Da. The results also showed that tetrandrine regulated the phosphorylation level of AKT by decreasing the oxidation of HSP90, along with coclaurine-*N*-gluA.

Together, these findings indicated that Hint1, ACP1, FGR, HSP90@157W + oxidation, and Prkca@220N + 845.4540 Da acted as the key targets of *S. tetrandra*, which showed therapeutic effects on RA by inhibiting the PI3K/Akt pathway. Also, tetrandrine, fangchinoline, corypamine, *N*-gluA-schefferine, Me,coclaurine-*O*-glc, coclaurine-*N*-gluA, and corydamine were found to be the major ingredients of *S. tetrandra* to act on these key targets of the PI3K/Akt pathway.

However, the study employed a dosage of STE up to 2.7 g/kg, which may not surpass a pharmacologically meaningful threshold. In designing the experiment, we initially calculated the administered dosage of STE based on general rules, such as those based on body surface areas (1.17 g/kg), differing from most pharmacological studies that applied doses exceeding 1 g/kg per day (Liang et al., 2010; Xu et al., 2023). Considering the contents of tetrandrine and fangchinoline in STE (Supplementary Table S1), the pre-experimental administration of STE reached up to 5.4 g/kg, but was ultimately determined to be a range of 0.35 g/kg to 2.7 g/kg for the study. This limitation underscores the importance of leveraging existing guidelines and standards in research involving medicinal plant extracts, as they notably elevate the reproducibility and precision of findings. Remarkably, valuable guidelines (Heinrich et al., 2020; Heinrich et al., 2022) provide a solid scientific foundation for experimental design and serve as a safeguard against common errors and biases, thereby enhancing the overall quality and reliability of the research undertaken.

## 5 Conclusion

This study was designed to determine the role of *S*. *tetrandra* in CIA rats, identify the regulators, and elucidate the mechanism underlying the therapeutic effects of the multiple ingredients of *S. tetrandra* on rheumatic disorders. To address this, a multi-omics joint analysis was performed to quantify the alterations in the endogenous metabolites, proteins, and PTMs in the PBMCs of the CIA rats treated with STE. The results showed that STE attenuated the inflammatory response and proliferation of PBMCs by mediating the key targets of the PI3K/Akt pathway, including Hint1, ACP1, FGR, HSP90, and Prkca. The cross-ome correlation analysis further evaluated the effect of each ingredient of *S. tetrandra* on these molecular targets. Consistent with previous findings regarding the inhibitory effects of tetrandrine and fangchinoline on the expression and phosphorylation of AKT, this study also confirmed that corypamine, corydamine, coclaurine-*N*-glucuronide, Me,coclaurine-*O*-glc, and *N*-gluA-schefferine can regulate the expression and phosphorylation of AKT, leading to the inactivation of the PI3K/Akt pathway. Overall, these findings highlighted the importance of investigating the anti-rheumatic effects of *S. tetrandra*, provided insights into the mechanism of action of *S. tetrandra*, and may act as a framework for future studies.

## Data Availability

The original contributions presented in the study are included in the article/Supplementary Material, further inquiries can be directed to the corresponding author/s..
