# Supplementary material for: Multi-omics analysis to reveal the synergistic mechanism underlying the multiple ingredients of Stephania tetrandra extract on rheumatoid arthritis through the PI3K/Akt signaling pathway
Source: Front Pharmacol. 2024 Aug 16;15:1447283. doi: 10.3389/fphar.2024.1447283 (PMC11361992; doi:10.3389/fphar.2024.1447283)
Supplement: Supplementary file 3 [file Table1.pdf]

Supplementary Table 1

兰州沃特莱斯生物科技有限公司

Lanzhou wotelaisi Biotechnology Co. , Ltd.

检验报告单

Certificate of Analysis

产品名称（Produce Name）:粉防己提取物

植物来源（Plant origin）:防已科植物粉防己 Stephania tetrandra S. Moore 的块根

批　　号（Batch Number）　:20180726　　　检验日期（Analysis Date）:2018-07-26

生产日期（Manufacture Date）:2018-07-26 失效日期（Expiry Date）:2020-07-25

批　　量（Batch Quantity）:500 公斤　　　使用部位（Used Part）：块根

检测项目<br>Items	标准<br>Standards	检测结果<br>Results
规格<br>Specifications	50:1	符合规定<br>Qualified
粉防己碱<br>Tetrandrine	1.34%	符合规定<br>Qualified
防己诺林碱<br>Fangchinoline	0.73%	符合规定<br>Qualified
性状<br>Description	棕色粉末<br>Brown powder	符合规定<br>Qualified
气味<br>Taste	特殊气味<br>Characteristic	符合规定<br>Qualified
粒径<br>Mesh Size	80 目<br>100％pass 80mesh	符合规定<br>Qualified
灼炽残渣<br>Residue on Ignition	≤10.0%	3.56％
干燥失重<br>Loss on Drying	≤5.0％	3.15％
重金属<br>Heavy Metal	≤10ppm	符合规定<br>Qualified
铅<br>（Pb）	≤2ppm	符合规定<br>Qualified
砷<br>（As）	≤2ppm	符合规定<br>Qualified
细菌总数<br>Total Plate Count	≤1000cfu/g	符合规定<br>Qualified
霉菌及酵母菌<br>Yeast&Mold	≤50cfu/g	符合规定<br>Qualified
大肠杆菌<br>E.coil	无<br>Negative	无<br>Negative
沙门氏菌<br>Salmonella	无<br>Negative	无<br>Negative

兰州沃特莱斯生物科技有限公司 质检专用章

质检科长：TRCT　　　　检验员：李易峰

Quality Assurance Office: TRCT　　　　Analyst:FengYiLee
