# Supplementary material for: Multi-omics analysis to reveal the synergistic mechanism underlying the multiple ingredients of Stephania tetrandra extract on rheumatoid arthritis through the PI3K/Akt signaling pathway
Source: Front Pharmacol. 2024 Aug 16;15:1447283. doi: 10.3389/fphar.2024.1447283 (PMC11361992; doi:10.3389/fphar.2024.1447283)
Supplement: Supplementary file 4 [file DataSheet1.pdf]

Supplementary Material

1.1 Supplementary Figures

Supplementary Figure 1. A: Chemical profile of *Stephania tetrandra* extract (Batch Number: 20180726, Lanzhouwotelaisi Bio-Tech Co., Ltd) by LC-HRMS/MS analysis. (1) Base peak chromatogram (BPC) of *S. tetrandra* Moore extract. (2) Base peak chromatogram (BPC) of *S. tetrandra* Yinpian (Tongrentang Chinese Medicine, Zhengzhou, China). (3) Extracted ion current chromatogram of corydamine. (4) Extracted ion current chromatogram of tetrandrine. (5) Extracted ion current chromatogram of Fangchinoline. B: Chemical structures of tetrandrine, fangchinoline, and corydamine.

Supplementary Figure 2. *Stephania tetrandra* extract (STE) suppressed the production of pro-inflammatory cytokines in serum of rats. The levels of IL-1β and IL-6 in serum of rats were determined by ELISA. Data are shown as the mean ± SD. * $p < 0.1$, ** $p < 0.05$, *** $p < 0.01$, vs. Mod group.

Supplementary Figure 3. Typical base peak chromatograms (BPCs) of serum samples from the CIA rats. (1) Representative chromatogram detected in positive ionization modes, using HILIC chromatography. (2) Representative chromatogram detected in negative ionization modes, using BEH chromatography. (3) Representative chromatogram detected in positive ionization modes, using T3 chromatography. (4) Representative chromatogram detected in positive ionization modes, using CSH chromatography.

Supplementary Figure 4. Principal component analysis was performed using quantitative values from metabolomic and lipidomic data (log2-transformed and centered around 0). (A) PCA score plot (principal component (PC) 1 versus PC2) of test samples and QC samples shows PC1 and 2 capture 33.73% and 18.01% of the variance between samples. (B) Metabolic pathways of the up regulated metabolites in STE rats' serum.

Supplementary Figure 5. The extracted ion current chromatogram (1) of the identified metabolites in the serum of rats after the oral administration of *Stephania tetrandra* extract, with the corresponding MS1 (2) and MS/MS spectra (3). (A) Tryptophan. (B) 5-Hydroxytryptophan. (C) Kynurenine. (D) 5-Hydroxyindoleacetic acid.

Supplementary Figure 6. The extracted ion current chromatogram (EIC, 1) and MS/MS spectra of ST-derived components in rats after the oral administration of *Stephania tetrandra* extract. (A) Fangchinoline. (B) Mecoclaurine-*O*-glc. (C) Corydamine. (D) Corypamine.

1.2 Supplementary Tables

Supplementary Table 1. Certificate of Analysis of *Stephania tetrandra* extract.

Supplementary Table 2. The datasets of dis-regulated metabolic pathways, related to Figure 4.

Supplementary Table 3. The datasets of Proteomics and PTMomics. (A) Normalized protein expression. (B) Normalized PTMomics PSMs. (C) Sample group of the datasets. The datasets were obtained as described in the methods..

Figure S1

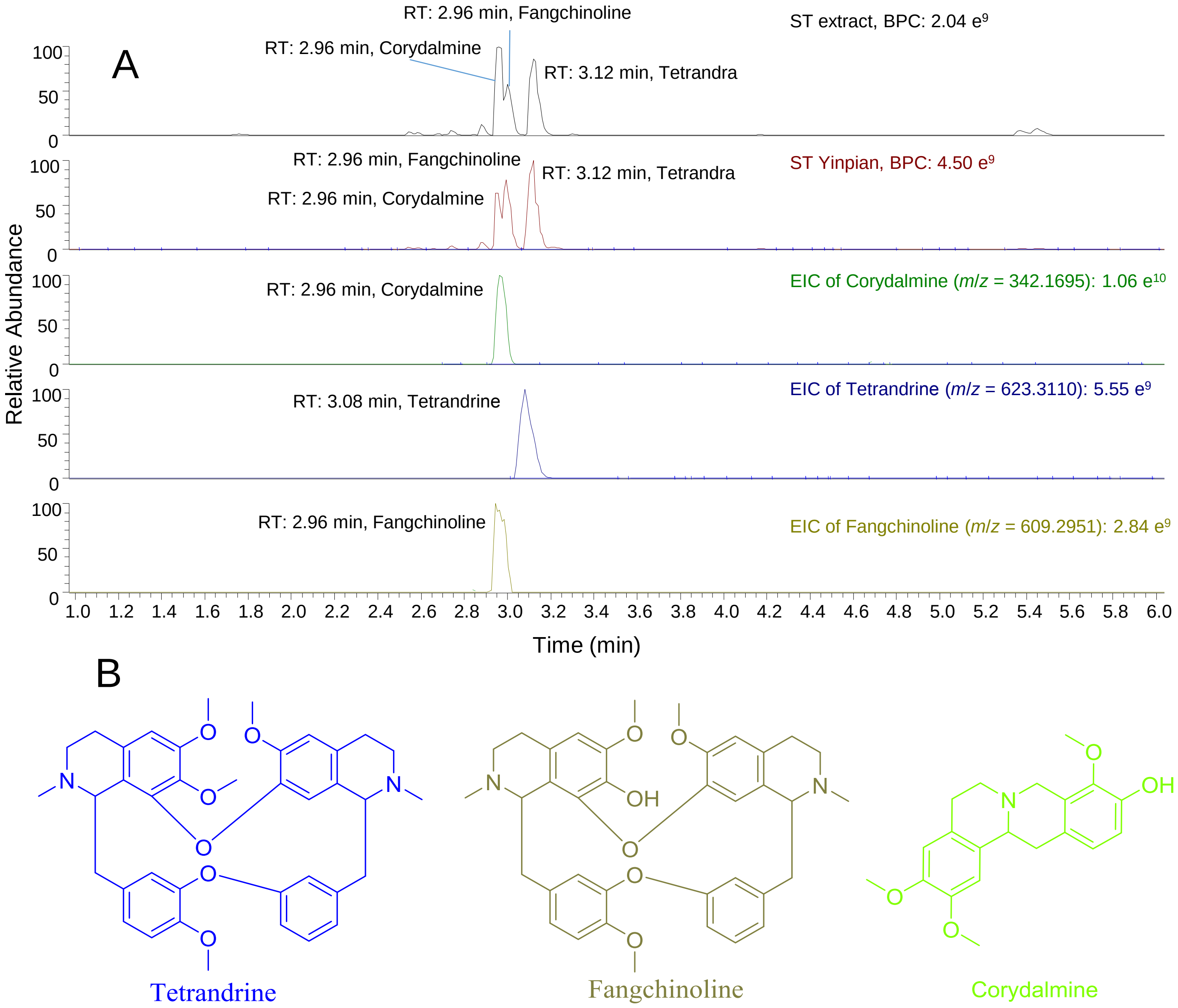

A
RT: 2.96 min, Fangchinoline
RT: 2.96 min, Corydalmine
RT: 3.12 min, Tetrandra
ST extract, BPC: 2.04 e9
ST Yinpian, BPC: 4.50 e9
EIC of Corydalmine (m/z = 342.1695): 1.06 e10
RT: 3.08 min, Tetrandrine
EIC of Tetrandrine (m/z = 623.3110): 5.55 e9
EIC of Fangchinoline (m/z = 609.2951): 2.84 e9
Relative Abundance
Time (min)
B
Tetrandrine
Fangchinoline
Corydalmine

Figure S2

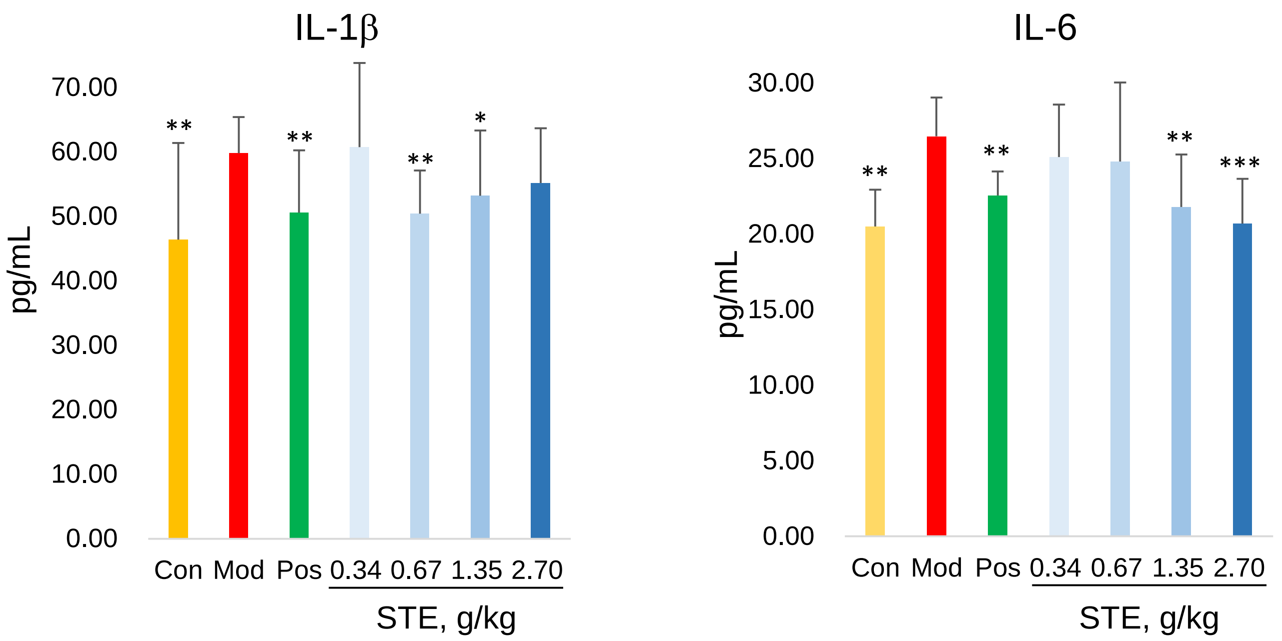

Figure S3

A

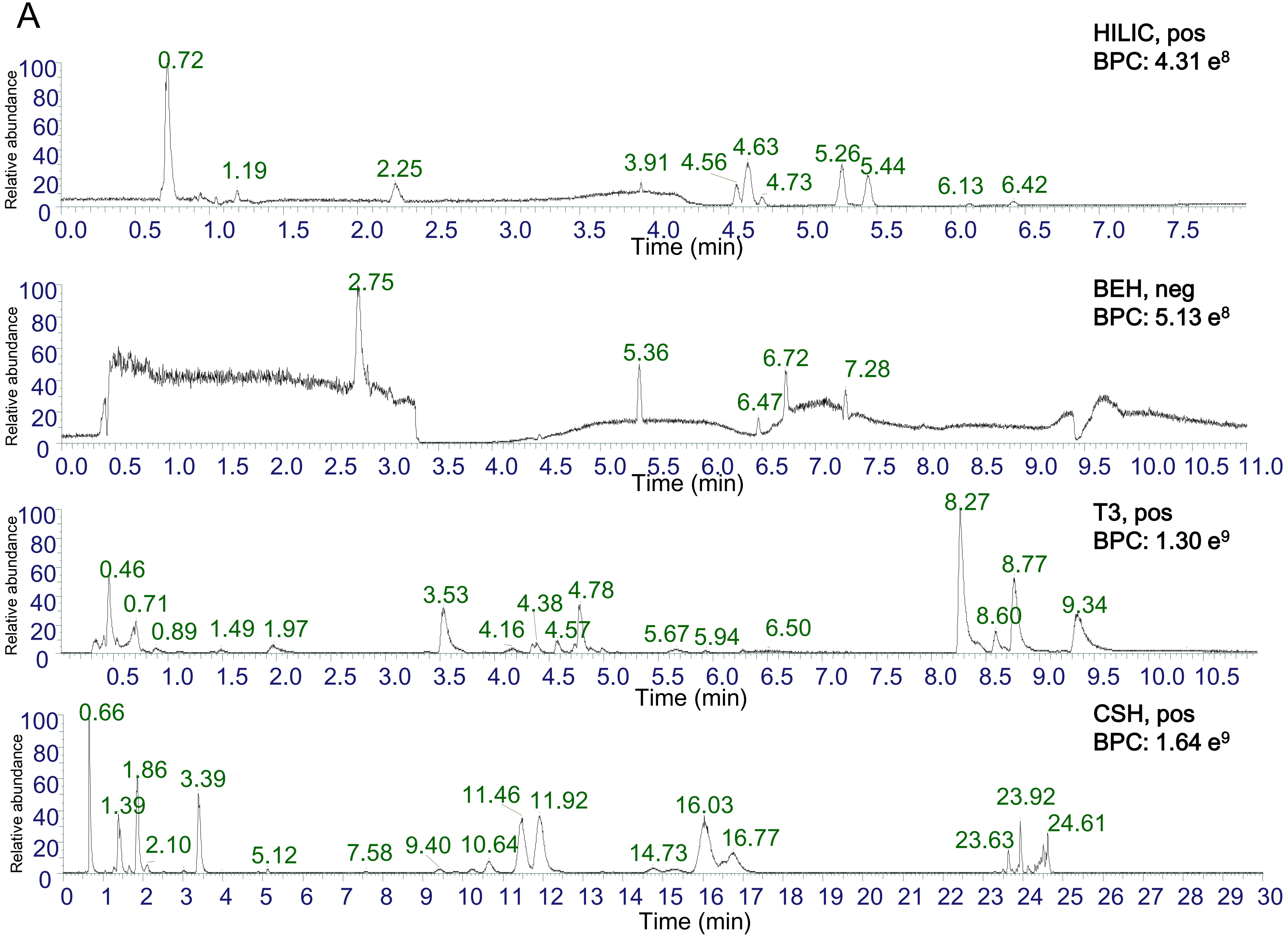

HILIC, pos
BPC: 4.31 e8
0.72
1.19
2.25
3.91
4.56
4.63
4.73
5.26
5.44
6.13
6.42
BEH, neg
BPC: 5.13 e8
2.75
5.36
6.47
6.72
7.28
T3, pos
BPC: 1.30 e9
0.46
0.71
0.89
1.49
1.97
3.53
4.16
4.38
4.57
4.78
5.67
5.94
6.50
8.27
8.60
8.77
9.34
CSH, pos
BPC: 1.64 e9
0.66
1.39
1.86
2.10
3.39
5.12
7.58
9.40
10.64
11.46
11.92
14.73
16.03
16.77
23.63
23.92
24.61
Relative abundance
Time (min)

Figure S4

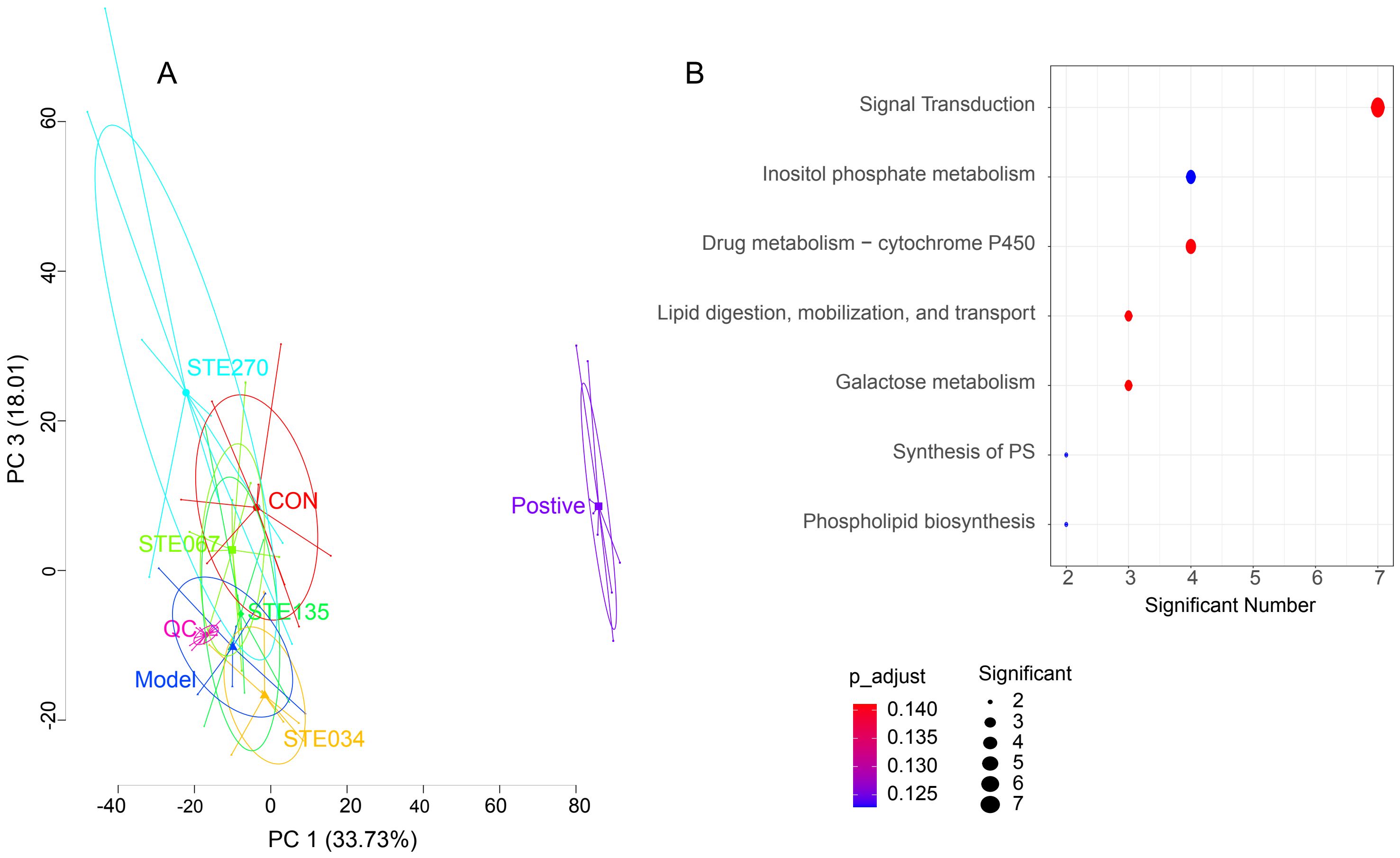
A
B
PC 3 (18.01)
PC 1 (33.73%)
STE270
CON
STE067
STE135
QC
Model
STE034
Postive
Signal Transduction
Inositol phosphate metabolism
Drug metabolism − cytochrome P450
Lipid digestion, mobilization, and transport
Galactose metabolism
Synthesis of PS
Phospholipid biosynthesis
Significant Number
p_adjust
0.140
0.135
0.130
0.125
Significant
2
3
4
5
6
7

Figure S5

A

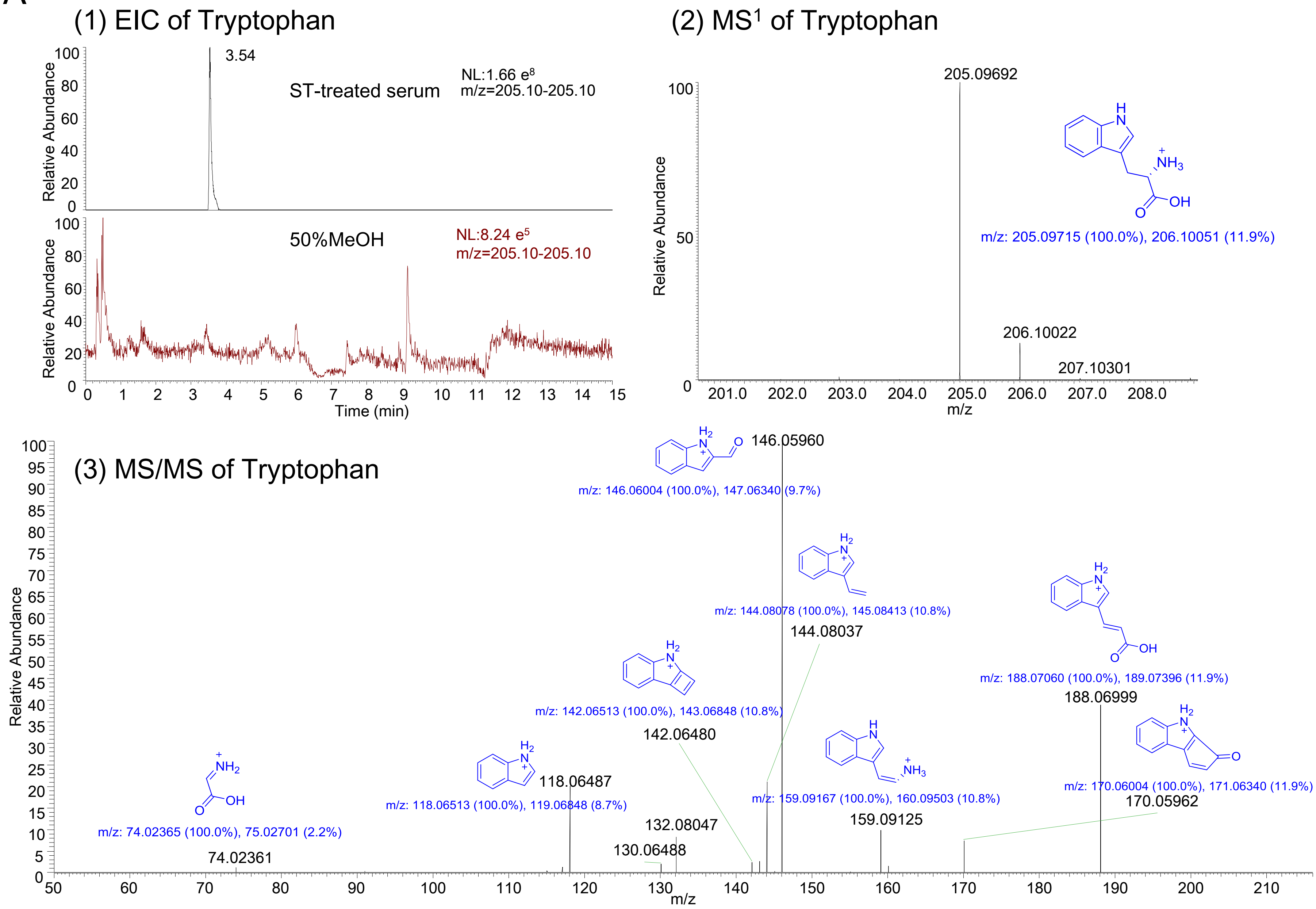

(1) EIC of Tryptophan
3.54
ST-treated serum
NL:1.66 e8
m/z=205.10-205.10
50%MeOH
NL:8.24 e5
m/z=205.10-205.10
Relative Abundance
Time (min)
(2) MS1 of Tryptophan
205.09692
206.10022
207.10301
m/z: 205.09715 (100.0%), 206.10051 (11.9%)
Relative Abundance
m/z
(3) MS/MS of Tryptophan
146.05960
m/z: 146.06004 (100.0%), 147.06340 (9.7%)
m/z: 144.08078 (100.0%), 145.08413 (10.8%)
144.08037
m/z: 188.07060 (100.0%), 189.07396 (11.9%)
188.06999
m/z: 142.06513 (100.0%), 143.06848 (10.8%)
142.06480
118.06487
m/z: 118.06513 (100.0%), 119.06848 (8.7%)
m/z: 159.09167 (100.0%), 160.09503 (10.8%)
159.09125
m/z: 170.06004 (100.0%), 171.06340 (11.9%)
170.05962
m/z: 74.02365 (100.0%), 75.02701 (2.2%)
74.02361
132.08047
130.06488
Relative Abundance
m/z

Figure S5B

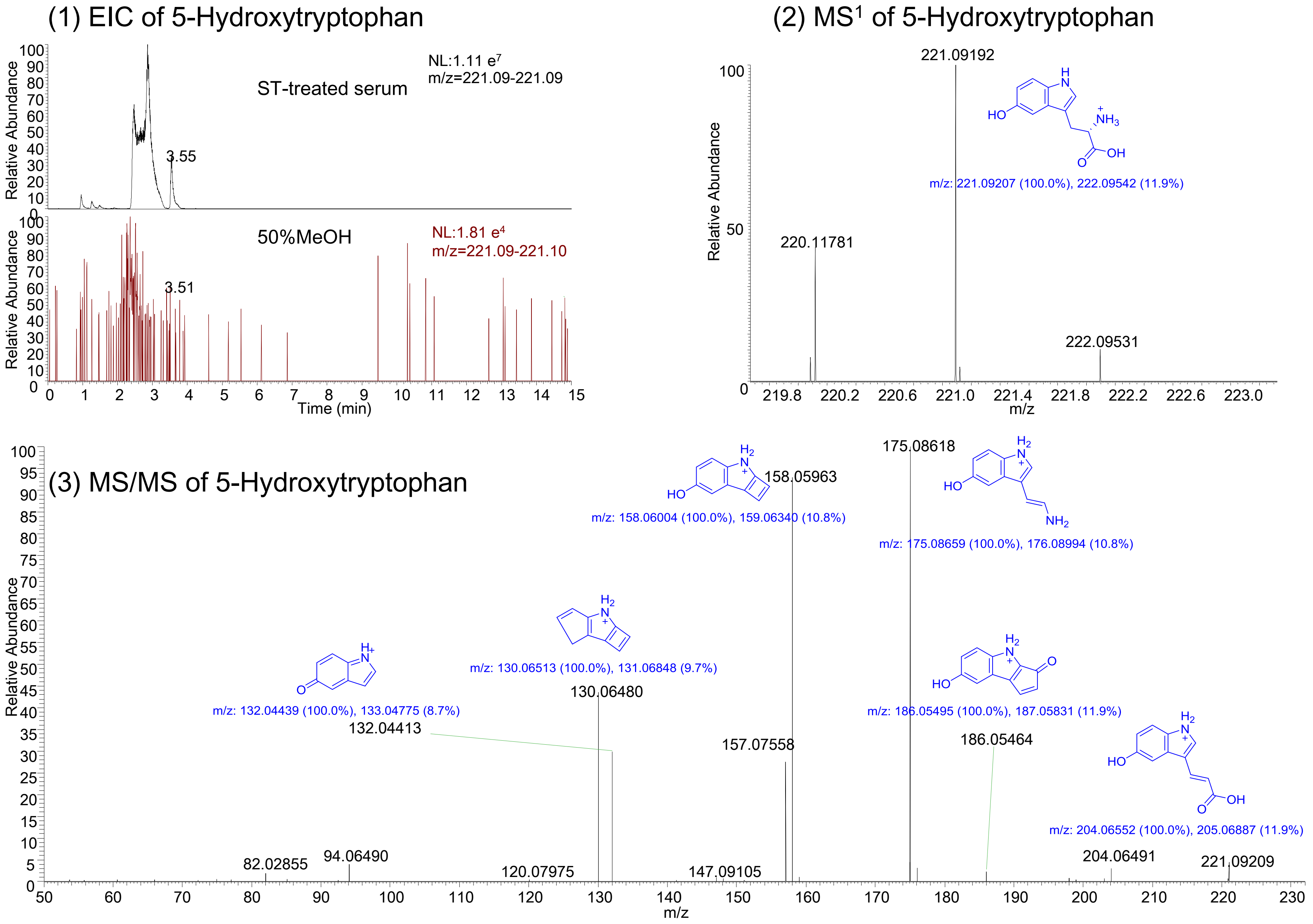
(1) EIC of 5-Hydroxytryptophan
ST-treated serum
NL:1.11 e7
m/z=221.09-221.09
3.55
50%MeOH
NL:1.81 e4
m/z=221.09-221.10
3.51
Relative Abundance
Time (min)
(2) MS1 of 5-Hydroxytryptophan
221.09192
220.11781
222.09531
m/z: 221.09207 (100.0%), 222.09542 (11.9%)
Relative Abundance
m/z
(3) MS/MS of 5-Hydroxytryptophan
175.08618
158.05963
m/z: 158.06004 (100.0%), 159.06340 (10.8%)
m/z: 175.08659 (100.0%), 176.08994 (10.8%)
m/z: 130.06513 (100.0%), 131.06848 (9.7%)
130.06480
m/z: 132.04439 (100.0%), 133.04775 (8.7%)
132.04413
157.07558
m/z: 186.05495 (100.0%), 187.05831 (11.9%)
186.05464
m/z: 204.06552 (100.0%), 205.06887 (11.9%)
204.06491
221.09209
82.02855
94.06490
120.07975
147.09105
Relative Abundance
m/z

Figure S5C

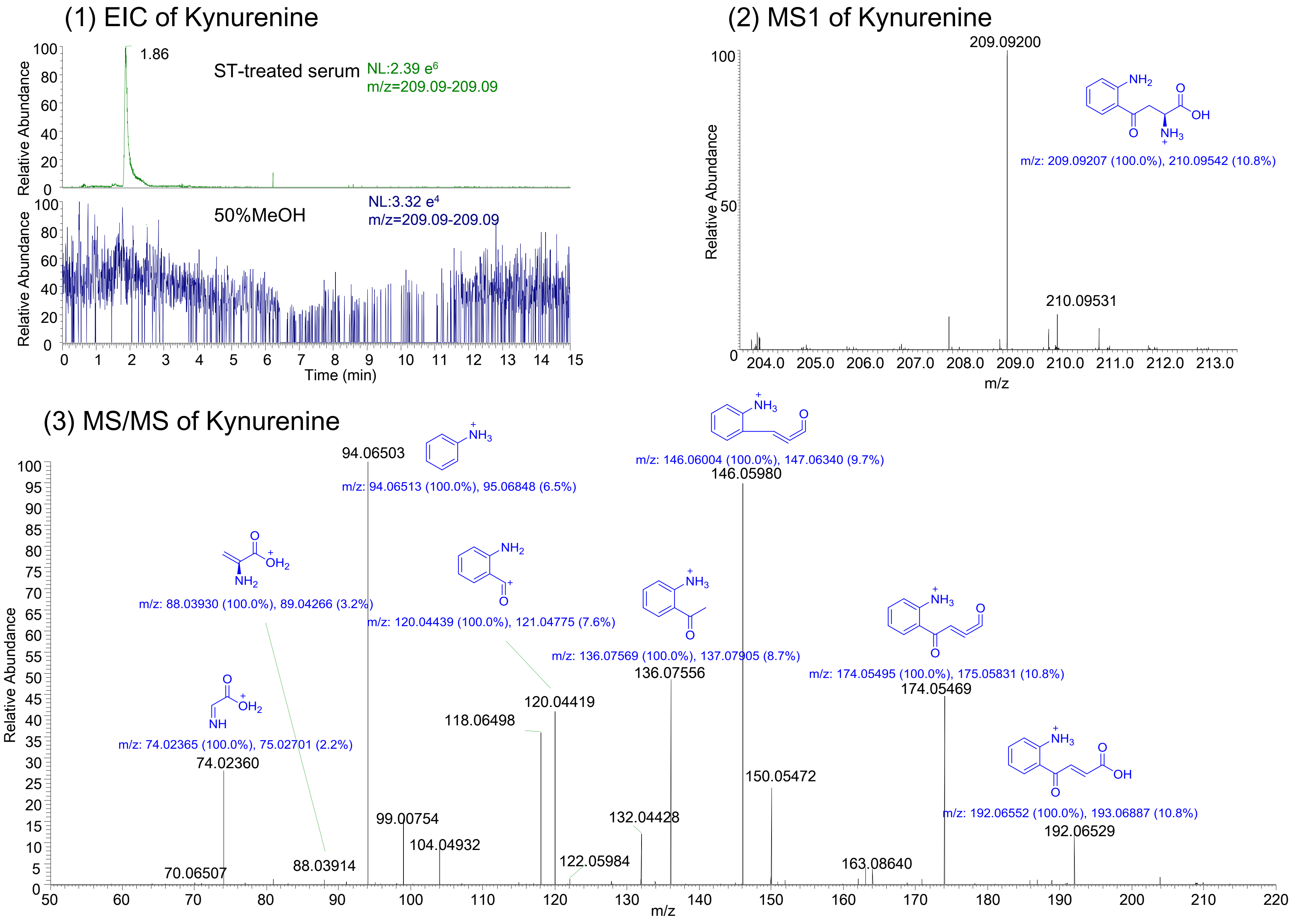

(1) EIC of Kynurenine
ST-treated serum
NL:2.39 e6
m/z=209.09-209.09
1.86
50%MeOH
NL:3.32 e4
m/z=209.09-209.09
Relative Abundance
Time (min)
(2) MS1 of Kynurenine
209.09200
210.09531
m/z: 209.09207 (100.0%), 210.09542 (10.8%)
Relative Abundance
m/z
(3) MS/MS of Kynurenine
94.06503
m/z: 94.06513 (100.0%), 95.06848 (6.5%)
146.05980
m/z: 146.06004 (100.0%), 147.06340 (9.7%)
m/z: 88.03930 (100.0%), 89.04266 (3.2%)
m/z: 120.04439 (100.0%), 121.04775 (7.6%)
m/z: 136.07569 (100.0%), 137.07905 (8.7%)
136.07556
m/z: 174.05495 (100.0%), 175.05831 (10.8%)
174.05469
120.04419
118.06498
m/z: 74.02365 (100.0%), 75.02701 (2.2%)
74.02360
150.05472
m/z: 192.06552 (100.0%), 193.06887 (10.8%)
192.06529
99.00754
132.04428
104.04932
122.05984
163.08640
88.03914
70.06507
Relative Abundance
m/z

Figure S5D

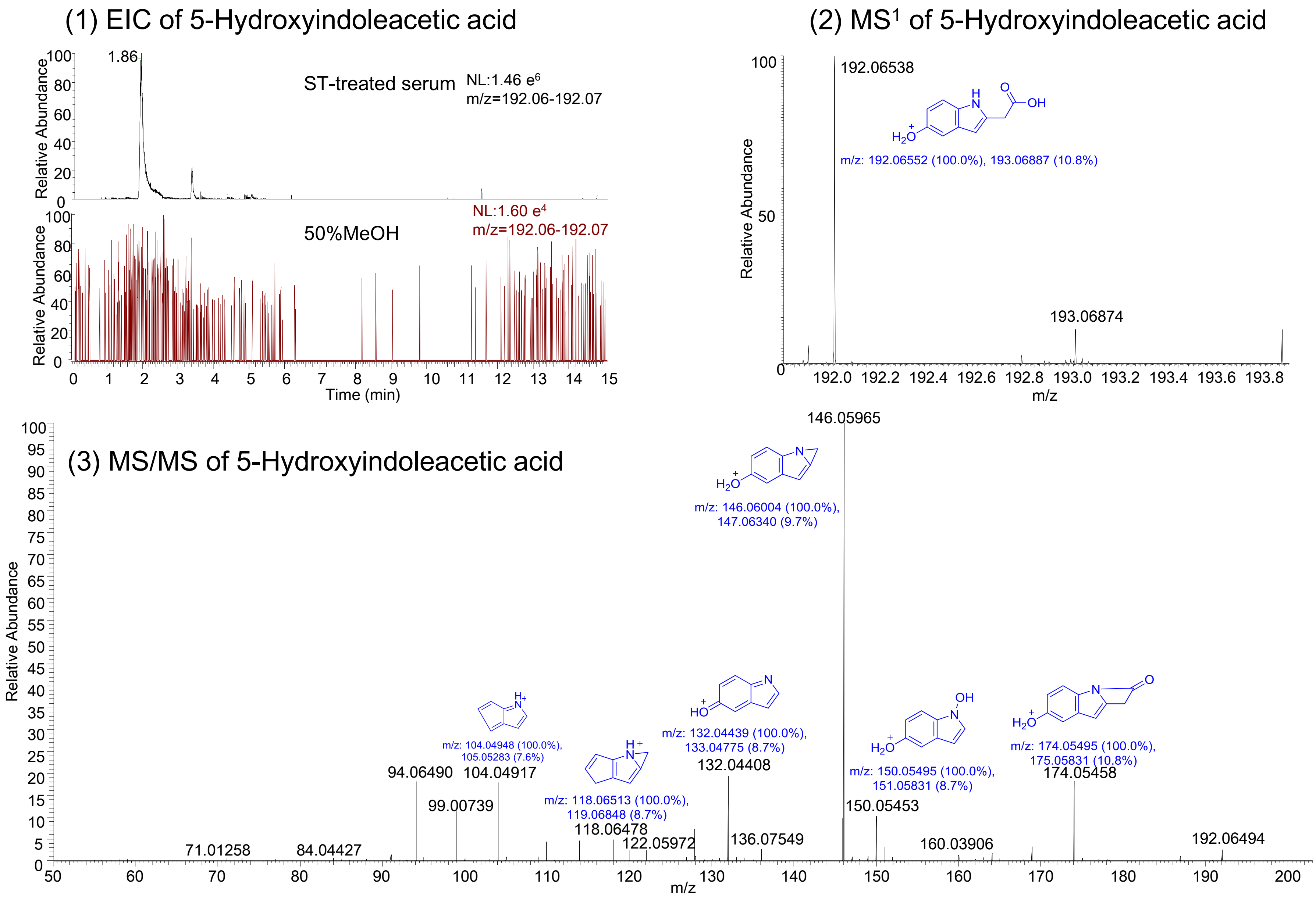

(1) EIC of 5-Hydroxyindoleacetic acid
(2) MS¹ of 5-Hydroxyindoleacetic acid
1.86
ST-treated serum
NL:1.46 e6
m/z=192.06-192.07
50%MeOH
NL:1.60 e4
m/z=192.06-192.07
Relative Abundance
Time (min)
192.06538
m/z: 192.06552 (100.0%), 193.06887 (10.8%)
193.06874
m/z
(3) MS/MS of 5-Hydroxyindoleacetic acid
146.05965
m/z: 146.06004 (100.0%), 147.06340 (9.7%)
m/z: 104.04948 (100.0%), 105.05283 (7.6%)
m/z: 118.06513 (100.0%), 119.06848 (8.7%)
m/z: 132.04439 (100.0%), 133.04775 (8.7%)
m/z: 150.05495 (100.0%), 151.05831 (8.7%)
m/z: 174.05495 (100.0%), 175.05831 (10.8%)
94.06490
104.04917
99.00739
118.06478
122.05972
132.04408
136.07549
150.05453
160.03906
174.05458
192.06494
71.01258
84.04427

Figure S6A

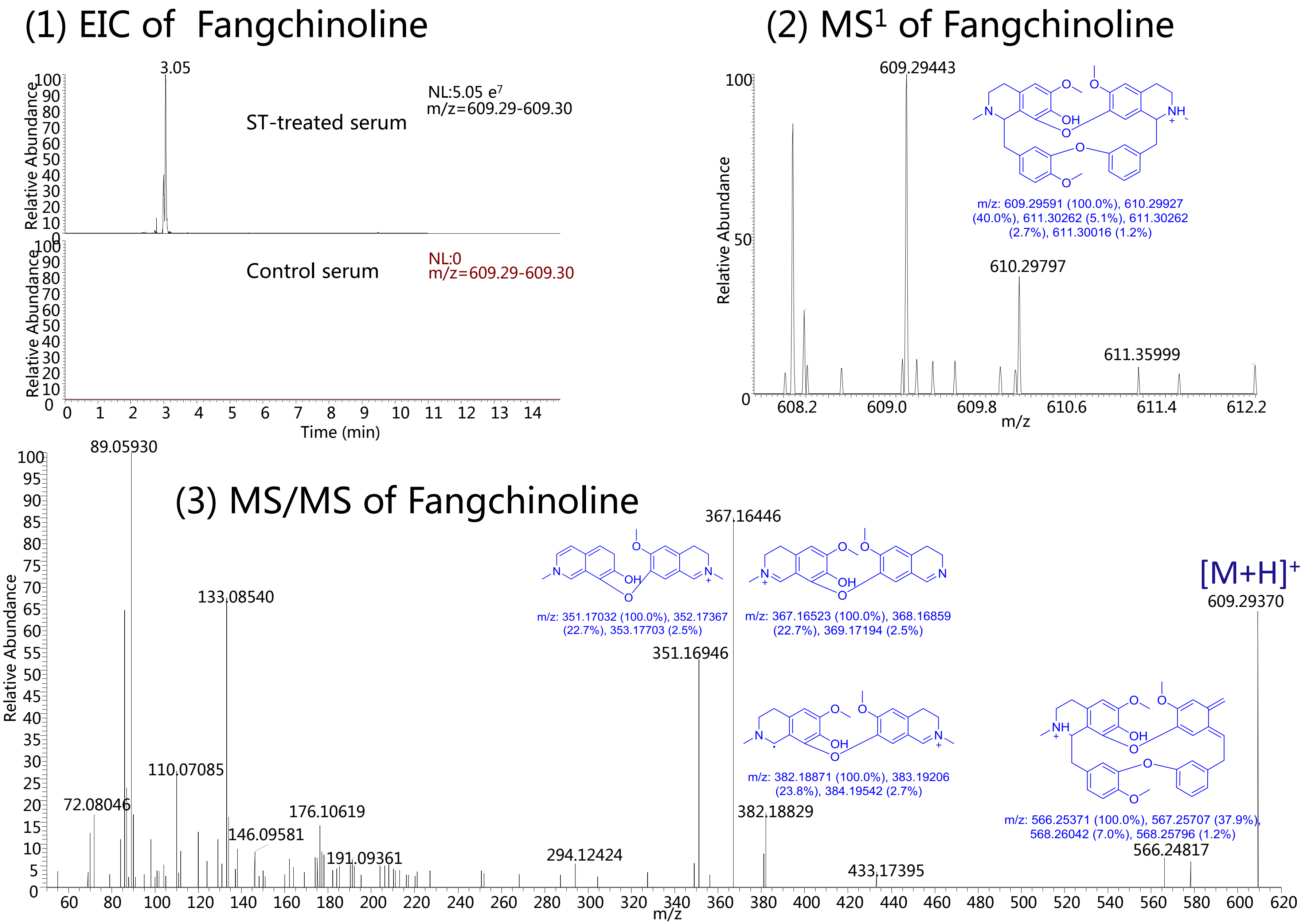
(1) EIC of Fangchinoline
3.05
NL:5.05 e7
m/z=609.29-609.30
ST-treated serum
Control serum
NL:0
m/z=609.29-609.30
Relative Abundance
Time (min)
(2) MS1 of Fangchinoline
609.29443
610.29797
611.35999
m/z: 609.29591 (100.0%), 610.29927 (40.0%), 611.30262 (5.1%), 611.30262 (2.7%), 611.30016 (1.2%)
Relative Abundance
m/z
(3) MS/MS of Fangchinoline
89.05930
133.08540
110.07085
72.08046
176.10619
146.09581
191.09361
294.12424
351.16946
367.16446
382.18829
433.17395
566.24817
[M+H]+
609.29370
m/z: 351.17032 (100.0%), 352.17367 (22.7%), 353.17703 (2.5%)
m/z: 367.16523 (100.0%), 368.16859 (22.7%), 369.17194 (2.5%)
m/z: 382.18871 (100.0%), 383.19206 (23.8%), 384.19542 (2.7%)
m/z: 566.25371 (100.0%), 567.25707 (37.9%), 568.26042 (7.0%), 568.25796 (1.2%)
Relative Abundance
m/z

Figure S6B

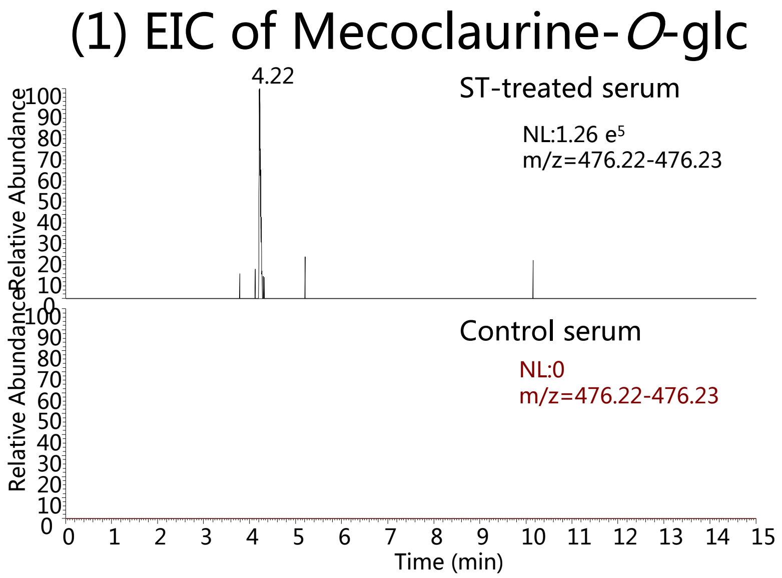
(1) EIC of Mecoclaurine-O-glc
4.22
ST-treated serum
NL:1.26 e5
m/z=476.22-476.23
Control serum
NL:0
m/z=476.22-476.23
Relative Abundance
Time (min)

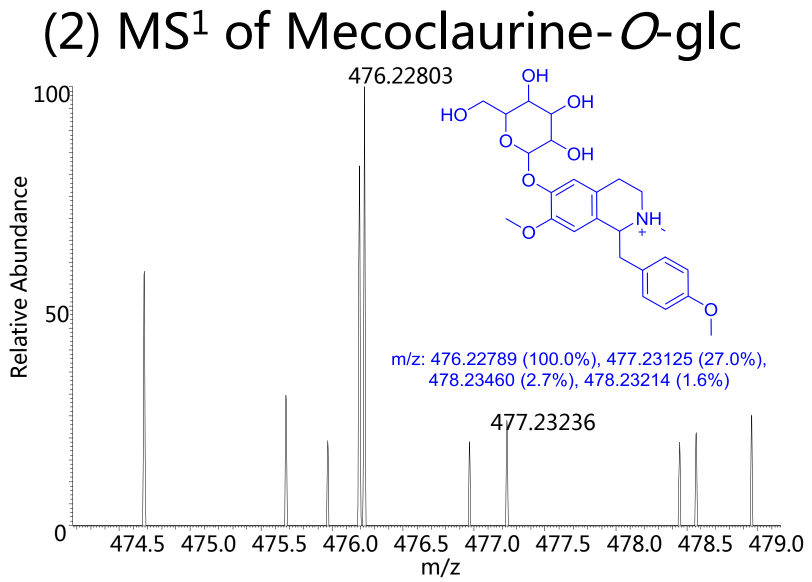
(2) MS1 of Mecoclaurine-O-glc
476.22803
477.23236
m/z: 476.22789 (100.0%), 477.23125 (27.0%), 478.23460 (2.7%), 478.23214 (1.6%)
Relative Abundance
m/z

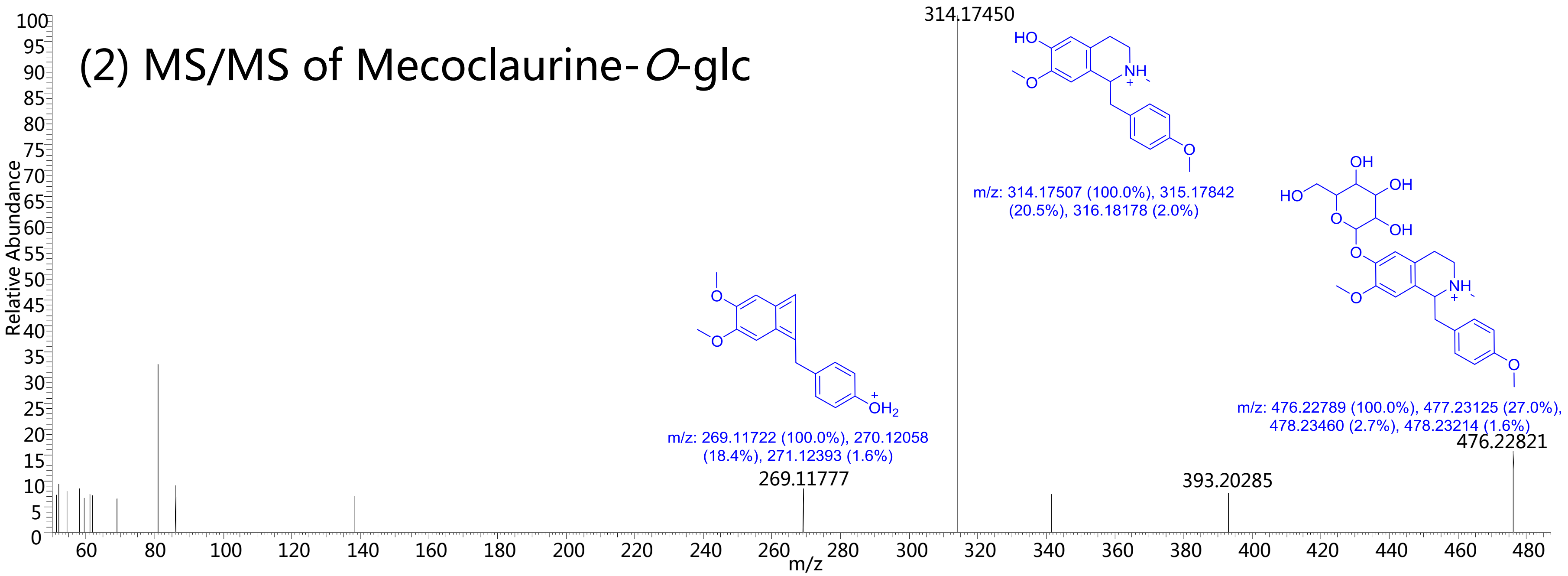
(2) MS/MS of Mecoclaurine-O-glc
314.17450
m/z: 314.17507 (100.0%), 315.17842 (20.5%), 316.18178 (2.0%)
m/z: 269.11722 (100.0%), 270.12058 (18.4%), 271.12393 (1.6%)
269.11777
393.20285
m/z: 476.22789 (100.0%), 477.23125 (27.0%), 478.23460 (2.7%), 478.23214 (1.6%)
476.22821
Relative Abundance
m/z

Figure S6C

(1) EIC of Schefferine/Corydamine

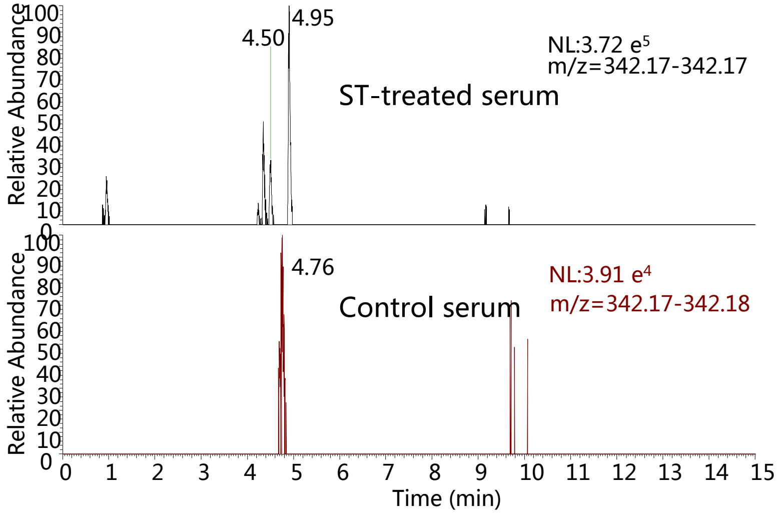

(2) MS^1 of Schefferine/Corydamine

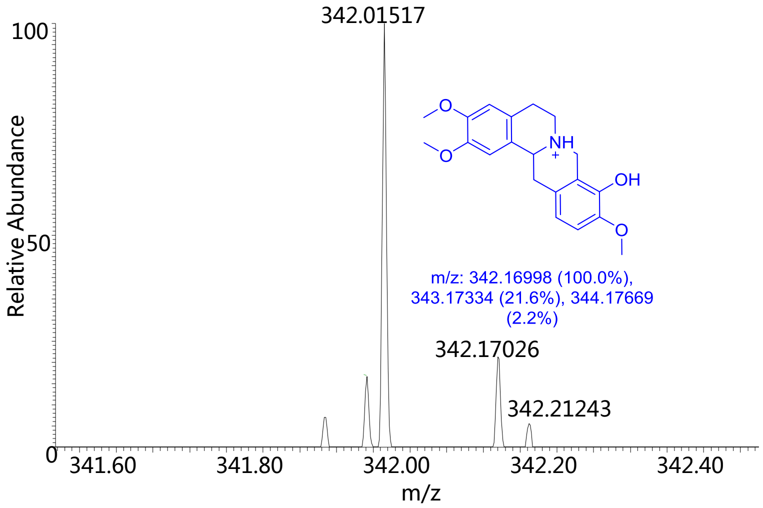

(3) MS/MS of Schefferine/Corydamine

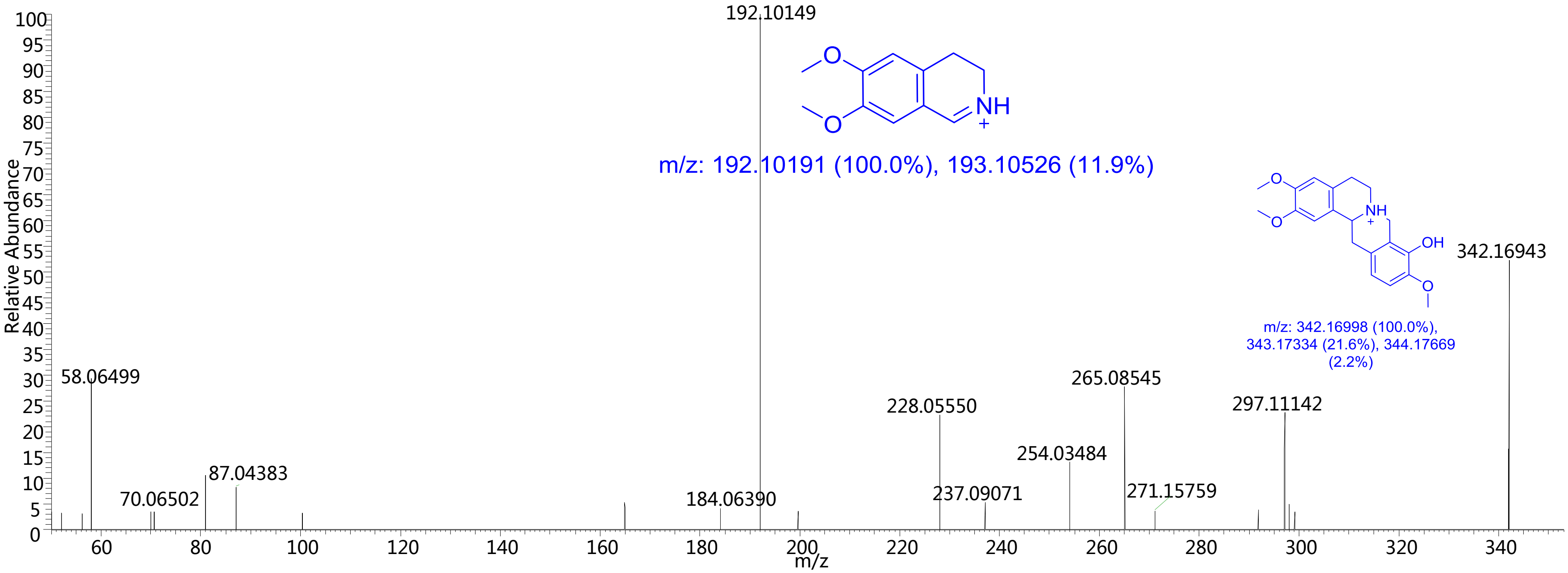

Figure S6D

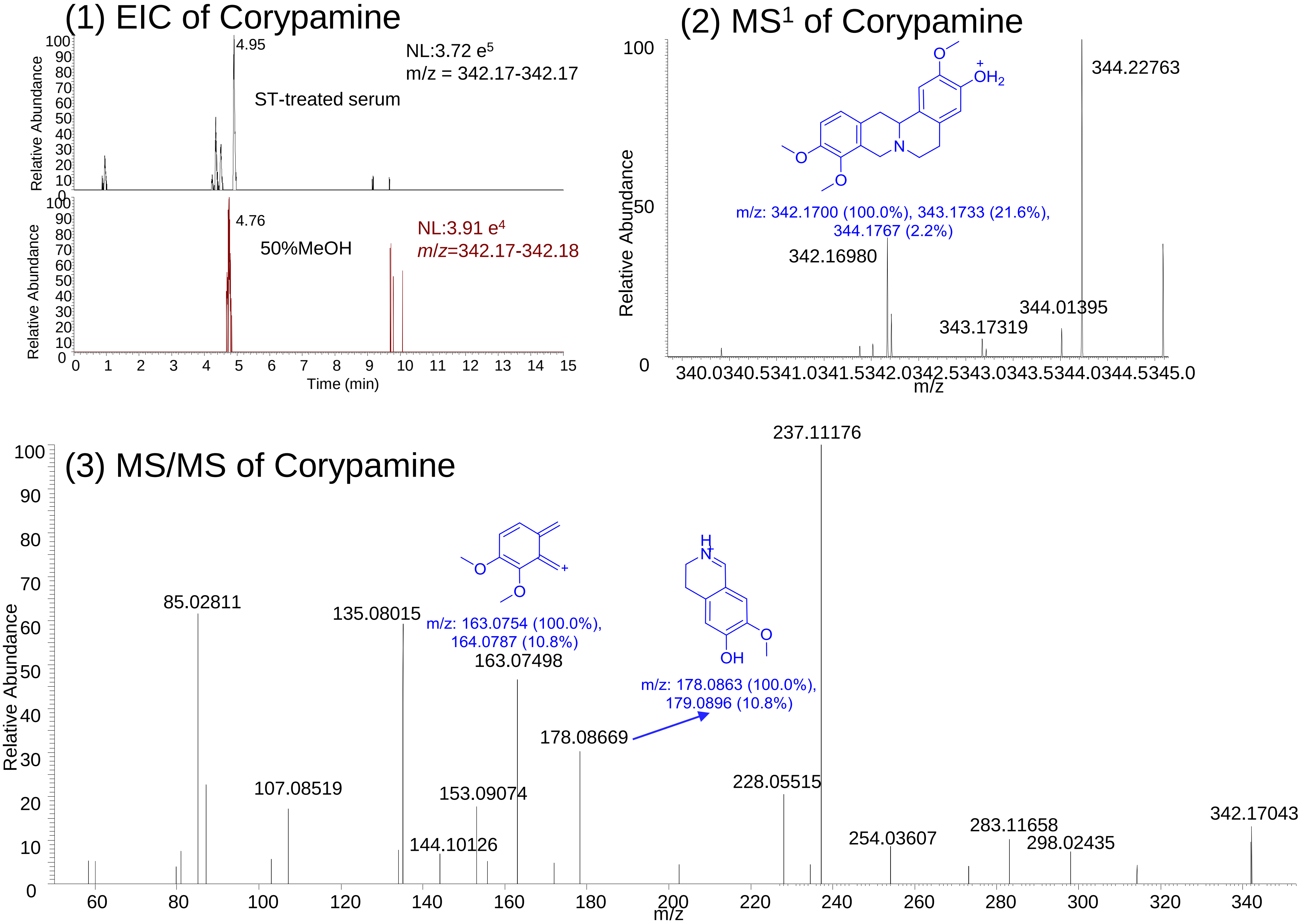
(1) EIC of Corypamine
Relative Abundance
4.95
NL:3.72 e5
m/z = 342.17-342.17
ST-treated serum
4.76
50%MeOH
NL:3.91 e4
m/z=342.17-342.18
Time (min)
(2) MS1 of Corypamine
344.22763
m/z: 342.1700 (100.0%), 343.1733 (21.6%), 344.1767 (2.2%)
342.16980
344.01395
343.17319
340.0340.5341.0341.5342.0342.5343.0343.5344.0344.5345.0
m/z
(3) MS/MS of Corypamine
237.11176
85.02811
135.08015
m/z: 163.0754 (100.0%), 164.0787 (10.8%)
163.07498
m/z: 178.0863 (100.0%), 179.0896 (10.8%)
178.08669
107.08519
153.09074
228.05515
144.10126
254.03607
283.11658
298.02435
342.17043
